# Comparative plastomes of *Carya* species provide new insights into the plastomes evolution and maternal phylogeny of the genus

**DOI:** 10.3389/fpls.2022.990064

**Published:** 2022-10-13

**Authors:** Jianwei Xi, Saibin Lv, Weiping Zhang, Jingbo Zhang, Ketao Wang, Haobing Guo, Jie Hu, Yang Yang, Jianhua Wang, Guohua Xia, Guangyi Fan, Xinwang Wang, Lihong Xiao

**Affiliations:** ^1^ State Key Laboratory of Subtropical Silviculture, Zhejiang A&F University, Hangzhou, China; ^2^ State Key Laboratory of Earth Surface Processes and Resource Ecology and Ministry of Education Key Laboratory for Biodiversity Science and Ecological Engineering, College of Life Sciences, Beijing Normal University, Beijing, China; ^3^ Department of Biological Sciences, St. John’s University - Queens, NY, United States; ^4^ The Beijing Genomics Institute (BGI) -Qingdao, The Beijing Genomics Institute (BGI)-Shenzhen, Qingdao, China; ^5^ Pecan Breeding and Genetics, Southern Plains Agricultural Research Center, USDA-ARS, College Station, TX, United States

**Keywords:** hickory, pecan, nut crop, EA-NA disjunction, structure diversity, plastome phylogeny

## Abstract

*Carya*, in the Juglandiodeae subfamily, is to a typical temperate-subtropical forest-tree genus for studying the phylogenetic evolution and intercontinental disjunction between eastern Asia (EA) and North America (NA). Species of the genus have high economic values worldwide for their high-quality wood and the rich healthy factors of their nuts. Although previous efforts based on multiple molecular markers or genome-wide SNPs supported the monophyly of *Carya* and its two EA and NA major subclades, the maternal phylogeny of *Carya* still need to be comprehensively evaluated. The variation of *Carya* plastome has never been thoroughly characterized. Here, we novelly present 19 newly generated plastomes of congeneric *Carya* species, including the recently rediscovered critically endangered *C. poilanei*. The overall assessment of plastomes revealed highly conservative in the general structures. Our results indicated that remarkable differences in several plastome features are highly consistent with the EA-NA disjunction and showed the relatively diverse matrilineal sources among EA *Carya* compared to NA *Carya*. The maternal phylogenies were conducted with different plastome regions and full-length plastome datasets from 30 plastomes, representing 26 species in six genera of Juglandoideae and *Myrica rubra* (as root). Six out of seven phylogenetic topologies strongly supported the previously reported relationships among genera of Juglandoideae and the two subclades of EA and NA *Carya*, but displayed significant incongruencies between species within the EA and NA subclades. The phylogenetic tree generated from full-length plastomes demonstrated the optimal topology and revealed significant geographical maternal relationships among *Carya* species, especially for EA *Carya* within overlapping distribution areas. The full-length plastome-based phylogenetic topology also strongly supported the taxonomic status of five controversial species as separate species of *Carya*. Historical and recent introgressive hybridization and plastid captures might contribute to plastome geographic patterns and inconsistencies between topologies built from different datasets, while incomplete lineage sorting could account for the discordance between maternal topology and the previous nuclear genome data-based phylogeny. Our findings highlight full-length plastomes as an ideal tool for exploring maternal relationships among the subclades of *Carya*, and potentially in other outcrossing perennial woody plants, for resolving plastome phylogenetic relationships.

## Introduction

The plastid, as a key uniparentally inherited organelle in plant cells, carries out not only photosynthesis but also other metabolic processes that mediate the adaptation of the plant to its environment (Dierckxsens et al., 2016). Despite increased interest in phylogenetic and phylogenomic analyses using nuclear genome datasets, plastome-based analyses have also become a powerful solution that have been widely applied to address recalcitrant relationships across the Tree of Life (Hu et al., 2016; Wei et al., 2017; Yan et al., 2018; Song et al., 2020; Wang et al., 2020; Tu et al., 2021; Dong et al., 2022; Liu et al., 2022; Ogoma et al., 2022). The plastome-based phylogenetic approaches play important roles in plant phylogenetics and evolution, such as revealing ancient and recent introgression or hybridization, tracking seed dispersal in phylogeographic studies, and charactering the structural diversity and evolution of organellar genomes (Zhao et al., 2018; Li et al., 2019; Yao et al., 2019; Li et al., 2020; Yang et al., 2021 and references therein). The most recent studies have produced numerous examples of phylogenetic discordance in plastomes or between plastids and nuclear gene trees at various evolutionary scales, which are interpreted as ancient introgressive hybridization, ancient chloroplast capture, or incomplete lineage sorting (Dong et al., 2022; Yang et al., 2021; Zhou et al., 2022). Some studies also emphasized the application of plastome-based analyses to track patterns of geographically structured interspecific gene flow, including several lineages of Fagales such as oak species with overlapping distribution ranges (Pham et al., 2017; Yang et al., 2021; Zhou et al., 2022).

Genus *Carya*, as the second largest genus in the Juglandiodeae subfamily of Fagales, contains typical temperate-subtropical forest trees for studying the phylogenetic evolution and intercontinental disjunction between eastern Asia (EA) and North America (NA) (Zhang et al., 2013; Huang et al., 2019). *Carya* is comprised of up to 20 extant species disjunctively distributed in EA and NA, and most species of the genera are also economically important for their valuable timbers and/or edible nut kernels (e.g., the pecan, Chinese hickory and Dabieshan hickory) (Grauke, 2003; Kozlowski et al., 2018; Huang et al., 2019; Xiao. et al., 2021). Based on their morphological characteristics, nineteen extant *Carya* species (excluding *C. dabieshanensis* that disputed as a variant of *C. cathayensis*) are divided into three sections based on morphological characters: section *Carya*, the true hickories (nine species); section *Apocarya*, the pecan hickories (four species); and section *Sinocarya*, the Asian hickories (six species) (Manning and Wayne, 1978; Grauke, 2003). The phylogeny and evolutionary history of *Carya* has been proposed by multiple studies based on extensive phylogenetic studies, combined with morphology, anatomy, cytology, and DNA sequences of nuclear and organelles (Manos and Stone, 2001; Manos et al., 2007; Zhang et al., 2013; Huang et al., 2019; Mu et al., 2020; Zhang et al., 2021). Zhang et al. (2013) investigated phylogenetic relationships between *Carya* species in EA and NA by integrating ten molecular markers from plastid (eight) and nuclear (two) with extensive taxon sampling, and they reconstructed the historical biogeography of *Carya* by integrating macro-fossil, morphological, and molecular data. However, the phylogeny among species in EA or NA subclades were hard to explain by any morphological traits (Zhang et al., 2013). Although the inference of biogeographical history gave more supports for the NA origin of *Carya* and the hypothetical migratory from NA to Europe and then to EA, evidence was not sufficient only based on a small set of molecular markers and partial macro-fossil records, in particular the omission of the early fossil records from the Kaliningrad region of former Soviet Union in Eurasia (Mai, 1981; Zhang et al., 2021). The most recent reports based on genome-wide SNPs (Huang et al., 2019), integrated RAD-seq and chloroplast genomes (Mu et al., 2020), and fossil-informed models provided strong support for backbone relationships among taxa of Juglandoideae, the monophyly of *Carya* genus, and its two major subclades in EA-NA (Zhang et al., 2021). However, significant inconsistencies were found among these studies phylogenetic topologies within EA or NA subclades, especially five taxonomically disputed species, *C. poilanei*, *C. sinensis* (the synonym name is *Annamocarya sinensis*), *C. dabieshanensis*, *C. glabra* and *C. ovalis*, that inferred from different molecular markers or datasets. Furthermore, *C. illinoinensis*, as the most commercially valuable species, has shown that many cultivars of this species were generated from intraspecies hybridization with other potential admixing species, such as *C. cordiformis*, *C. aquatica* and *C. myristiciformis* (Lovell et al., 2021). Recently, based on the pan-genome assemblies of pecan genotypes and resequencing data of multiple genotypes above potential admixing species, several disease-related interspecific genomic introgression blocks have been identified in the genotypes of *C. illinoinensis* (Lovell et al., 2021). However, the maternal relationships of these pecan varieties with different economically important traits have never been addressed. Meanwhile, the maternal phylogeny of *Carya* still need to be comprehensively evaluated, and the variation of *Carya* plastome has never been thoroughly characterized.

*Carya poilanei* (Chev.), also known as Poilane’s hickory, had three original collections in Vietnam, Laos, and Thailand (Chevalier, 1941; Leroy, 1950; Manning, 1963; Grauke et al., 1991). This rare species was initially described as *Juglans poilanei* (Chevalier, 1941). It was suspected to be extinct in the wild since the 1950s when Leroy placed the species under *Carya* (Leroy, 1950; Leroy, 1955; Grauke et al., 1991; Grauke et al., 2016). Until most recently, three subpopulations were rediscovered in the Ailao Mountain, Yunnan province, China, and *C. poilanei* was instead suggested to be listed as critically endangered (Zhang et al., 2022). Thus, the phylogenetic relationship of *C. poilanei* with other *Carya* species needs to be clarified. *Carya sinensis* (Dode), with a common name of ‘Hui He Tao’ (i.e. beaked walnut or beaked hickory) in China and ‘Cay Cho Dai’ in Vietnam, is narrowly distributed in southern China and northern Vietnam (Chevalier, 1941; Manning and Hjelmqvist, 1951). The species was first described by Dode (1912) and categorized as a separate genus *Annamocarya indochinensis* (Chevalier, 1941). It was subsequently ascribed to six different genera: *Annamocarya*, *Rhamphocarya*, *Juglandicarya*, *Caryojuglans*, *Juglans*, and *Carya* (Leroy, 1950; Manning and Hjelmqvist, 1951; Scott, 1953; Leroy, 1955; Lu et al., 1999). Although the previous and recent reports based on the molecular markers evidenced the taxon of the tree as a member of section *Sinocarya* in EA *Carya*, discordance remains among the phylogenetic topologies that were built based on different data sets (Zhang et al., 2013; Luo et al., 2021). *Carya dabieshanensis* M. C. Liu & Z. J. Li., historically considered a variant of *C. cathayensis*, is now treated as an addition to the nomenclature of Chinese hickory (Liu and Li, 1984). However, collections of germplasm and voucher specimens for more thorough comparison are necessary, and the relationship between *C. dabieshanensis* and *C. cathayensis* still needs to be addressed (Manos and Stone, 2001). Distribution maps of the widely distributed NA species, *Carya glabra* (Mill.) Sweet. with the common name pignut hickory, have included *C. ovalis* (Wangenh.) Sarg. (common name red hickory) since the reduction of *C. ovalis* to synonymy with *C. glabra* (Little, 1969). Although *C. glabra* readily hybridized with *C. ovalis* when the two occurred together, with hybrids confusing the distinctions between the species (Manning, 1950), the morphological characteristics and their habitats show that they are separate species in the section *Carya* in NA (Manning, 1950; Grauke, 2003). Nevertheless, *C. glabra* and some other species in NA *Carya* were also wrongly identified as other species partially because of the interspecific hybridization in nature stands (Heiges, 1896). These circumstances, together with the inconsistent results based on molecular inferences (Zhang et al., 2013; Huang et al., 2019), make them imperative for a more comprehensive study on the phylogeny of *Carya* and the development of molecular markers for species identification. *Carya illinoinensis* (Wangenheim) K. Koch (common name pecan), as a member of the section *Apocarya*, is native to the United States and Mexico, with over 8,000 years of history (Hester, 1983; Grauke et al., 2011; Wang et al., 2020). The latest microsatellite profiles revealed ‘87MX3-2.11’ to be homozygous (Wang et al., 2020). Owing to the richness in health factors of its nut kernels, *C. illinoinensis* became the most commercially valuable species of *Carya*. Commercial production of *C. illinoinensis* has persisted for about a century and a half, and it has been widely planted across six continents with more than 400 cultivars released so far, including some varieties promoted and planted in large areas (Grauke et al., 2016; Xiao. et al., 2021). Among the cultivars of *C. illinoinensis*, many were formed by both natural and artificial hybridization (https://cgru.usda.gov/CARYA/PECANS). For example, the varieties ‘Pawnee’, ‘Lakota’ and ‘Elliott’ mentioned above, belong to intraspecies hybrids with interspecies introgression in history, based on whole genome sequencing data (Thompson et al., 2008; Huang et al., 2019; Lovell et al., 2021; Xiao et al., 2021; https://cgru.usda.gov/CARYA/PECANS). However, the maternal relationships of the varieties are still unclear, the investigation of plastome variations inherited from the female parent may provide important clues for tracking both ancient and recent gene flows among different varieties caused by inter- and intraspecies hybridization.

In this study, we newly assembled and characterized the structures and diversity of complete plastomes for the critically endangered *C. poilanei* and other 18 congeneric species, based on the sequences, generated by high-throughput sequencing technology. To explore the optimal phylogenetic relationship within *Carya*, the phylogenetic trees were reconstructed respectively by employing the entire or partial plastome sequences and unique protein-coding sequences of 30 plastomes representing all 20 *Carya* species (19 newly assembled, plus the recent release of *C. pallida*), plus six representatives from five other genera of Juglandoideae. The maternal relationship between *C. dabieshanensis* and *C. cathayensis* was determined by integrating the phylogenetic relationship with the comparison of morphological features, and confirmed the taxonomic status of *C. dabieshanensis*, *C. cathayensis*, *C. sinensis*, *C. glabra*, and *C.,poilanei* as separate species in section *Sinocarya*. The maternal relationships and discordance of species in EA and NA *Carya*, and several varieties in *C. illinoinensis* were also discussed based on the reconstruction of plastome phylogenetic topologies of *Carya*. The results generated here are of great value for the evolution, wild resource conservation and genetic breeding of *Carya* in the future. Our methodology of exploring the phylogenetic relationships of *Carya* species in EA or NA, by using the different molecular markers or the informative fragments of plastomes with different evolutionary rates, will provide a good example for the reconstruction of biogeography under subgenus in future, especially for outcrossing perennial tree species.

## Materials and methods

### Plant materials

In this study, fresh, fully expanded young leaves from an adult tree of *C. poilanei* (**Supplementary Figure 1**) were collected in July from the eastern edge of the Ailao Mountains in Jianshui County, in the southern Yunnan Province of China. Fresh, fully expanded young leaves from *C. illinoinensis* (cv. ‘Sioux’), *C. cathayensis*, *C. dabieshanensis C. hunanensis*, and *C. tonkinensis* were collected from an orchard in Zhejiang A&F University, China. The samples of fresh fully expanded young leaves from *C. kweichowensis* and *C. sinensis* were collected from the Qiannan Buyi and Miao Autonomous Prefecture, Guizhou Province, China, respectively. All collected leaf samples were immediately immersed in liquid nitrogen for transportation back to the laboratory and stored at -80°C before DNA extraction. The details of the collected samples are listed in **Supplementary Table 1**.

The trunk, branches, leaves, flowers and fruits of *C. dabieshanensis* and *C. cathayensis* planted in the orchard of Zhejiang A&F University, Hangzhou, China, were photographed and characterized by their morphologic traits. Meanwhile, the nuts of other EA *Carya* species were also photographed.

### DNA extraction and sequencing

The high-quality genomic DNA was isolated from approximately 100 mg samples using the E.Z.N.A.^®^ HP Plant DNA Mini kit (Omega Bio-Tek, USA). DNA quality and concentration were assessed in a Qubit 3.0 Fluorometer (Thermo Fisher Scientific Inc., USA), and DNA integrity was evaluated by a 1.0% (W/V) agarose gel.

Approximately 1μg of high-quality genomic DNA from each sample was used for the whole genome sequencing (WGS). The high-throughput sequencing library of *C. poilanei* was constructed and sequenced using the Illumina NovaSeq 6000 platform (NovoGene, Beijing, China) following the standard procedure of the manufacturer. The MGIEasy DNA Rapid Library Prep Kit (cat.# 1000006985; MGI-Tech., China) was used to construct the sequencing library of *C. illinoinensis* (cv. ‘Sioux’), *C. cathayensis*, *C. dabieshanensis*, *C. hunanensis*, *C. tonkinensis*, *C. kweichowensis* and *C. sinensis*, and the paired-end 100 bp reads were generated on the BGISEQ-500 platform (BGI, Shenzhen, China) according to the manufacturers’ procedures, respectively. The raw sequence data was submitted to the Sequence Read Archive (SRA) of NCBI (**Supplementary Table 1**).

### Data processes, assembly, validation and annotation

To obtain the high-quality plastome assemblies of 19 *Carya* species, 2 Gb raw reads were randomly selected for each species from NovaSeq 6000 data (*C. poilanei*), BGISEQ-500 PE100 data (seven species that were sequenced in this study) or from Illumina HiSeq X-TEN data retrieved from the NCBI database (eleven previously sequenced species), respectively. The low-quality reads from all samples, i.e., reads having over 50% bases with a quality value below 12, adaptor only and more than 10% N bases, were filtered out by SOAPnuke software (Chen et al., 2018), separately. The trimmed clean reads including nuclear and organelle genome data were used to assemble the plastomes for each species. The plastomes of 19 *Carya* species were assembled *via* NOVOPlasty software (version 3.7), with ‘Seed_RUBP_cp.fasta’ provided by the software as the seed input and the published *Juglans regia* plastome (accession no. NC_028617.1) as reference (Dierckxsens et al., 2016; Peng et al., 2017). The default K-mer size (K-mer = 39) was firstly selected and multiple K-mers were used for a synchronous assembling test during the assembling to obtain the ideal assemblies.

To verify the correction of the plastome assemblies, PCR amplification and Sanger sequencing were performed in the six EA species and the pecan variety ‘Sioux’, to further confirm the SC-IR boundaries of the assembled sequences, as well as several special genomic regions among EA species. The sequences of the primers are listed in **Supplementary Table 2**.

The plastome assemblies were annotated using the online program GeSeq – Annotation of Organellar Genomes (Michael et al., 2017). Initial annotation, putative starts, stops, and intron positions were predicted against the annotation of *Juglans regia* and *C. illinoinensis* (GenBank accession numbers: NC_028617.1 and NC_041449.1) plastomes. The final annotations were determined by integrating the GeSeq prediction and manual correction. The circular plastome maps of *Carya* species were constructed using the online visualization program Organellar Genome DRAW version 1.3.1 (OGDRAW) (Stephan et al., 2019). The newly generated complete plastome sequences were deposited in GeneBank (Accession numbers were listed in **Supplementary Table 3**).

### Comparative analyses of plastome structure features

The online program IRscope (Amiryousefi et al., 2018) was used to visualize the boundaries of LSC-IRb, IRb-SSC, SSC-IRa, and IRa-LSC of plastome among the 19 *Carya* species, The IR expansion and contraction among these *Carya* species were also compared afterward. DnaSP v. 6.12.03 (Julio et al., 2017) was employed to analyze the nucleotide diversities, sequence polymorphisms, and relative rates of plastome sequence divergence in the eighteen *Carya* species. In order to calculate the synonymous (*Ks*) and non-synonymous (*Ka*) substitution rates and the nucleotide variance (*π* and *θ*), we extracted the same individual functional protein-coding exons and aligned them separately using BioEdit v. 7.0.9.0 (Hall, 1999). To obtain selection patterns in protein-coding genes, we calculated the *Ka* and *Ks* values of each protein coding gene between two plastomes in 19 *Carya* species using DnaSP v. 6.12.03, and divided them to take the average to get the *Ka/Ks* ratio of each gene.

The online tool MIcroSAtellite (MISA, http://pgrc.ipk-gatersleben.de/misa/) was used to discover simple sequence repeats (SSRs or microsatellites) in the eighteen plastomes with the following parameters: ten for mono-nucleotide motifs, six for di-nucleotide, five for tri-nucleotide, and three for tetra-, penta- and hexa-nucleotides, respectively (Sebastian et al., 2017). The repeat numbers in the regions of LSC, SSC, IRa, and IRb were counted.

Codon usage bias was calculated using MEGA X v. 10.1.8 (Kumar et al., 2018) and EMBOSS v.6.3.1 (Peter et al., 2000; Itaya et al., 2013). In general, many genes less than 300 bp in length are believed have no real functions and the encoded proteins may not be accurate and do not necessarily contain domains. Too many such genes may interfere with the accuracy of the results. Therefore, the protein-coding genes with more than 300 nucleotides in length were extracted according to the annotation file of each plastome.

RNA-editing sites of protein-coding genes in *Carya* plastomes were predicted using the online program Predictive RNA Editor for Plant cp genes (PREP-Cp) suite with a minimal editing score of 0.7 (Mower, 2009) http://prep.unl.edu/).

### Phylogenetic analyses

To construct the phylogenomic trees, 30 plastome sequences were included, including 23 plastomes from 20 species in genus *Carya* (19 newly assembled *Carya* species, 2 C*. pallida* varieties and 2 published *C. illinoinensis* varieties), 6 representative species from 5 other genera in Juglandoideae (*Juglans regia*, *J. sigillata*, *Cyclocarya paliurus*, *Engelhardia roxburghiana*, *Platycarya strobiacea*, and *Pterocarya stenoptera*), and the *Myrica rubra* in Myricaceae (as outgroups). In addition, the plastome sequences of seven other plastome assemblies were downloaded from GenBank (Supplementary Table 3). Supplementary Table 3 lists all taxa used in the phylogenomic analyses, including their sampling locations and GenBank accession numbers.

The phylogenetic relationships were inferred based on seven datasets from all 30 plastomes: the full-length sequences of plastomes; the sequences of LSC, SSC, or IR regions; the 79 concatenated protein-coding sequences (CDSs); and the LSC-SSC or LSC-IR-SSC ZZconcatenated sequences. Multiple sequence matrices of the dataset were generated in MAFFT v7.490 under standard parameters (Kazutaka and Standley, 2013), and manually adjusted, respectively. The phylogenetic trees were reconstructed based on Bayesian inference (BI) using MrBayes v3.2.6 (Fredrik et al., 2012), and the maximum-likelihood (ML) method using PHYLIP version 3.698 (http://evolution.genetics.washington.edu/phylip.html). For BI inference, two independent Markov chain Monte Carlo (MCMC) simulations were run for 2,000,000 generations and sampled every 100 generations. MrBayes settings for the best-fit model (GTR+I+G) were selected by AIC in MrModeltest. The first 25% of calculated trees were discarded as burn-in. A consensus tree and Bayesian posterior probabilities (PPBI) were constructed using the remaining trees. The ML trees were reconstructed using in PHYLIP v.7.490 and the bootstrap value was set to 500. Both BI and ML trees were visualized in FIGTREE v1.4.3 (http://tree.bio.ed.ac.uk/software/figtree/).

To facilitate our analyses, we generated a map of modern distribution for each *Carya* species. Meanwhile, we also generated a map by combining the distributions and the diversity of IR-SC boundaries with the sampling location of each analyzed *Carya* species using the ArcGIS v10.2 program (Esri. ArcGIS software on https://www.esri.com/).

## Results

### General characteristics of *Carya* plastomes

**Figure 1:**
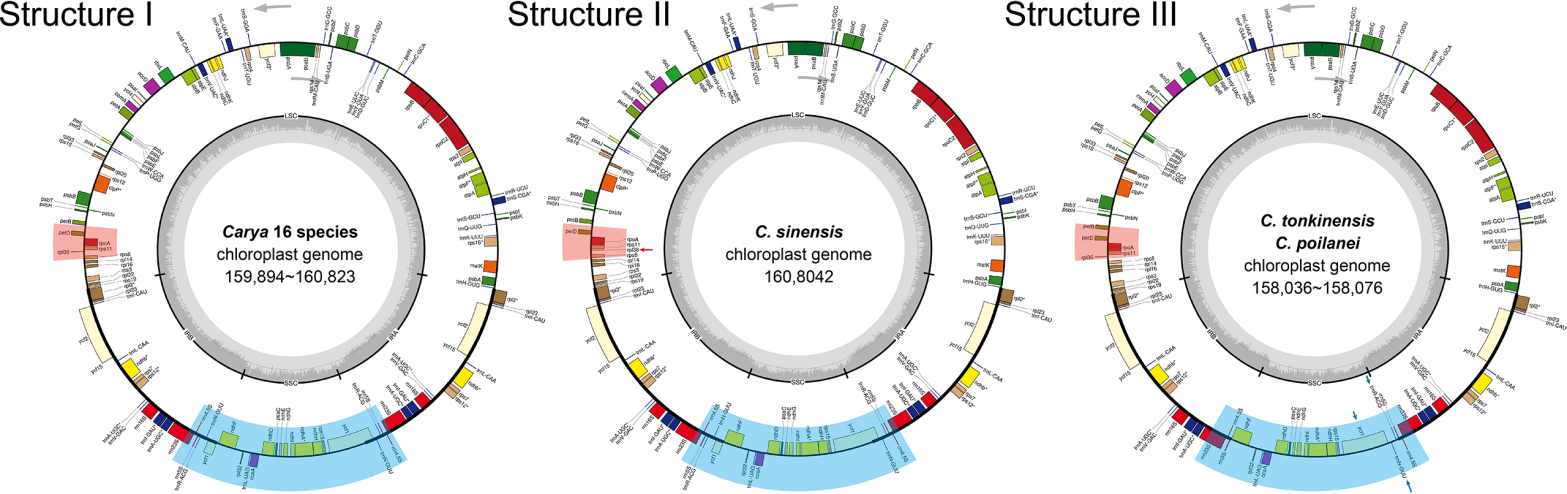
Circular maps of the 19 *Carya* species plastomes. GC content graphs are included as dark gray bars toward the center of each diagram. Intron-containing genes are marked with (*). Gray arrows indicate the translation direction of protein-coding genes. I - III show the different structures of *Carya* plastomes. Areas with light red and light blue backgrounds show differences in structure, and detailed differences are indicated by red and blue arrows.

Using 2-Gb whole-genome resequencing data from each species, we *de-novo* assembled the complete plastomes of 19 *Carya* species with the *J. regia* chloroplast genome as reference (Peng et al., 2017). Boundaries between the regions of inverted repeats (IRs) and single copies (SCs) of the assemblies were verified by PCR (**Supplementary Figures 2, 3**; **Supplementary Table 2**). Sequence alignment of the amplificated sequences with our assemblies displayed a perfect match, indicating our assembly’s accuracy and the inaccuracy of the assembly (additional duplication in SSC region with ca. 15 kb in length) of the published *C. kweichowensis* plastome sequence (Ye et al., 2018). Like those previously published plastomes in *Juglans* and *Carya* (Dong et al., 2017; Hu et al., 2017; Peng et al., 2017; Feng et al., 2018; Ye et al., 2018; Zhai et al., 2019; Wang et al., 2020), all the newly assembled *Carya* plastomes were circular molecules ca. 160 kb in length on average with the typical quadripartite structure, i.e., a pair of inverted repeat (IR) regions, separated by a large single-copy (LSC) region and a small single-copy (SSC) region (**Figure 1**; **Supplementary Figure 2**). Among the 19 plastomes of *Carya*, *C. poilanei* has the shortest sequence length (158,036 bp), followed by *C. tonkinensis* (158,076 bp), while *C. cathayensis* (160,823 bp) has the longest genome (**Table 1**). *C. cathayensis* has the longest LSC region (90,114 bp) but *C. hunanensis* has both the shortest LSC (89,468 bp) and SSC regions (18,730 bp). *C. palmeri* has the largest IR region (26,004 bp), whilst *C. poilanei* has the smallest IR region (23,634 bp) and the largest SSC region (20,861 bp). GC content do not display significant variation among the 19 *Carya* species (36.13% ~ 36.28%), as well as in the regions of LSC (33.71% ~ 33.87%), SSC (29.66% ~ 32.61%) and IRs (42.57% ~ 42.89%) among the 19 *Carya* plastomes assemblies (**Table 1**). However, the paired-IR regions have the highest GC content, because of the presence of four pairs of duplicated *rRNAs* in the IR regions of each species (**Table 1**).

**Table 1:** Summary of complete plastome features of the 19 newly assembled *Carya* plastomes.

Species	Length (bp)				GC content (%)				Unique genes					Total genes^d^
	Total	LSC	SSC	IRa/b	Total	LSC	SSC	IRa/b	Total	Protein coding	Intron containing^a^	tRNA^b^	rRNA^c^	
*C. cathayensis*	160,823	90,114	18,761	25,974	36.13	33.71	29.74	42.63	113	79	14	30	4	131
*C. dabieshanensis*	160,233	89,509	19,056	25,834	36.18	33.82	29.66	42.67	113	79	14	30	4	131
*C. hunanensis*	159,894	89,468	18,730	25,848	36.24	33.84	30.02	42.66	113	79	14	30	4	131
*C. kweichowensis*	160,223	89,846	18,731	25,823	36.22	33.79	30.08	42.68	113	79	14	30	4	131
*C. sinensis*	160,042	89,723	18,735	25,792	36.28	33.87	30.06	42.73	113	79	14	30	4	131
*C. tonkinensis*	158,076	89,940	20,860	23,638	36.15	33.76	31.19	42.88	113	79	14	30	4	129
*C. poilanei*	158,036	89,907	20,861	23,634	36.16	33.78	32.61	42.89	113	79	14	30	4	129
*C. aquatica*	160,763	89,966	18,791	26,003	36.15	33.75	29.89	42.58	113	79	14	30	4	131
*C. cordiformis*	160,793	89,989	18,798	26,003	36.15	33.74	29.88	42.58	113	79	14	30	4	131
*C. floridana*	160,760	89,964	18,792	26,002	36.14	33.75	29.89	42.58	113	79	14	30	4	131
*C. glabra*	160,652	89,888	18,786	25,989	36.20	33.80	30.02	42.57	113	79	14	30	4	131
*C. illinoinensis* ‘Sioux’	160,714	89,917	18,791	26,003	36.16	33.76	29.89	42.58	113	79	14	30	4	131
*C. laciniosa*	160,787	89,921	18,864	26,001	36.16	33.79	29.81	42.58	113	79	14	30	4	131
*C. myristiciformis*	160,788	89,990	18,792	26,003	36.15	33.74	29.89	42.58	113	79	14	30	4	131
*C. ovalis*	160,822	90,032	18,786	26,002	36.15	33.74	29.89	42.58	113	79	14	30	4	131
*C. ovata*	160,727	89,930	18,809	25,994	36.19	33.78	29.87	42.58	113	79	14	30	4	131
*C. palmeri*	160,672	89,886	18,778	26,004	36.17	33.78	29.88	42.57	113	79	14	30	4	131
*C. texana*	160,745	89,964	18,793	25,994	36.17	33.77	29.90	42.58	113	79	14	30	4	131
*C. tomentosa*	160,784	89,988	18,792	26,002	36.15	33.75	29.89	42.58	113	79	14	30	4	131

a-d, the number includes only one copy if the genes are located in the IR regions.

The 19 *Carya* complete plastomes have the same amounts of unique genes (a total of 113 unique genes including 79 conserved protein-coding genes, 30 *tRNAs*, and 4 *rRNAs*) and introns, which were arranged with a similar gene order (**Figure 1**; **Table 1**). Fourteen of the unique genes were intron-containing genes (9 protein-coding genes and 5 *tRNA* genes), with 1 or 2 introns (**Table 2**; **Supplementary Table 4**). Five intron-containing genes (3 protein-coding genes and 4 *tRNA* genes) are located within the IR regions (**Supplementary Table 4**). However, the intron size varied among the *Carya* plastomes, except for *ndhB*, *rps12*, *trnA-UGC*, *trnL-UAA* and *trnV-UAC*. The length of introns in the plastomes ranged from 526 bp to 1,229 bp and the longest intron was observed in *ndhA*. Two protein-coding genes, *rpl2* and *ycf3*, exhibit significant variations in the length of both exons and introns, with considerable differences among species from EA and NA, so as the *rpoC1* and *trnA-UGC* in their intron regions.

**Table 2:** Genes and contents in the newly assembled *Carya* plastomes.

Category	Group	Genes and number		
		LSC	SSC	IRa/b^a^
Photosynthesis	Subunits of photosystem I	*psaA, psaB, psaI, psaJ* (4)	*psaC* (1)	–
	Subunits of photosystem II	*psbA, psbB, psbC, psbD, psbE, psbF, psbH, psbI, psbJ, psbK, psbL, psbM, psbN, psbT, psbZ* (15)	–	–
	Subunits of NADH dehydrogenase	*ndhC, ndhJ, ndhK* (3)	ndhA^*,^ ndhD, ndhE, ndhF, ndhG, ndhH, ndhI (7)	ndhB^*^ (1X2)
	Subunits of cytochrome b/f complex	*petA, petB, petD, petG, petL, petN* (6)	–	–
	Subunits of ATP synthase	atpA, atpB, atpE, atpF*, atpH, atpI (6)	–	–
	Large subunit of Rubisco	*rbcL* (1)	–	–
				
Self-replication	Large subunits of ribosome	*rpl14, rpl16, rpl20, rpl22, rpl33, rpl36* (6)	*rpl32* (1)	*rps7, rps12* ^*^^d^ (2X2)
	Small subunits of ribosome	rps2, rps3, rps4, rps8, rps11, rps14, rps16*, rps18, rps19 (9)	*rps15* (1)	*rpl2* ^*^ *, rpl23* (2X2)
	DNA-dependent RNA polymerase	rpoA, rpoB, rpoC1*, rpoC2 (4)	–	–
	Ribosomal RNAs	–	–	*rrn16S, rrn23S, rrn4.5S, rrn5S* (4X2)
	Transfer RNAs	trnC-GCA, trnD-GUC, trnE-UUC, trnF-GAA, trnfM-CAU, trnG-GCC, trnH-GUG, trnK-UUU, trnL-UAA^*^, trnM-CAU, trnP-UGG, trnQ-UUG, trnR-UCU, trnS-CGA^*^, trnS-GCU, trnS-GGA, trnS-UGA, trnT-GGU, trnT-UGU, trnV-UAC^*^, trnW-CCA, trnY-GUA (22)	*trnL-UAG* (1)	trnA-UGC^*^, trnI-CAU, trnI-GAU^*^, trnL-CAA, trnN-GUU ^b^ *, trnR-ACG* ^b^ *, trnV-GAC* (7X2)
				
Other genes	Maturase	*matK* (1)	–	–
	Protease	clpP* (1)	–	–
	Envelope membrane protein	*cemA* (1)	–	–
	Acetyl-CoA carboxylase	*accD* (1)	–	–
	C-type cytochrome synthesis gene	–	*ccsA* (1)	–
				
Hypothetical reading frames	Proteins of unknown function	ycf3^*^, ycf4 (2)	*ycf1* ^c^ (1)	*ycf15, ycf2* (2X2)

a, located in IR regions with two copies; b, *trnR-ACG* and *trnN-GUU* only located in SSC region in *C. tonkinensis* and *C. poilanei* with single copy; c, *ycf1* gene located in SSC region for *C. tonkinensis* and *C. poilanei*, but the most part of its genic region located in SSC and small part in IRa region for other species; d, the most part of rps12 located in IR regions and only small part in LSC region; * indicated intron-containing gene.

### Structural variations among *Carya* plastomes

Annotation-based circular maps of the 19 *Carya* plastomes can be categorized into three distinct structures (**Figure 1**). The major structure (Structure I) is shared by 16 *Carya* plastomes, with the same number of genes (131 genes, representing 113 unique genes), gene order, and translation direction (**Figure 1**). Eighteen unique genes were duplicated in the IR regions of these 16 plastomes (**Table 2**). Structure II is the most different from the major structure (Structure I) with respect to the direction of transcription of *rpl36*, which uses the complement strand as a transcriptional template in *C. sinensis* (**Figure 1**). A sequence loss of ~2.3 kb in length in the IRb region of *C. poilanei* and *C. tonkinensis* plastomes leads to distinguished genome structure (Structure III) and decreased gene content (a total of 129 genes). The loss of 2.3 kb sequences results in a contracted IR region and the loss of *tRNA* genes, *trnN-GUU*, and *trnR-ACG* in the IRb region (**Figure 1**; **Table 2**).

### IR junction variations among *Carya* plastomes

**Figure 2:**
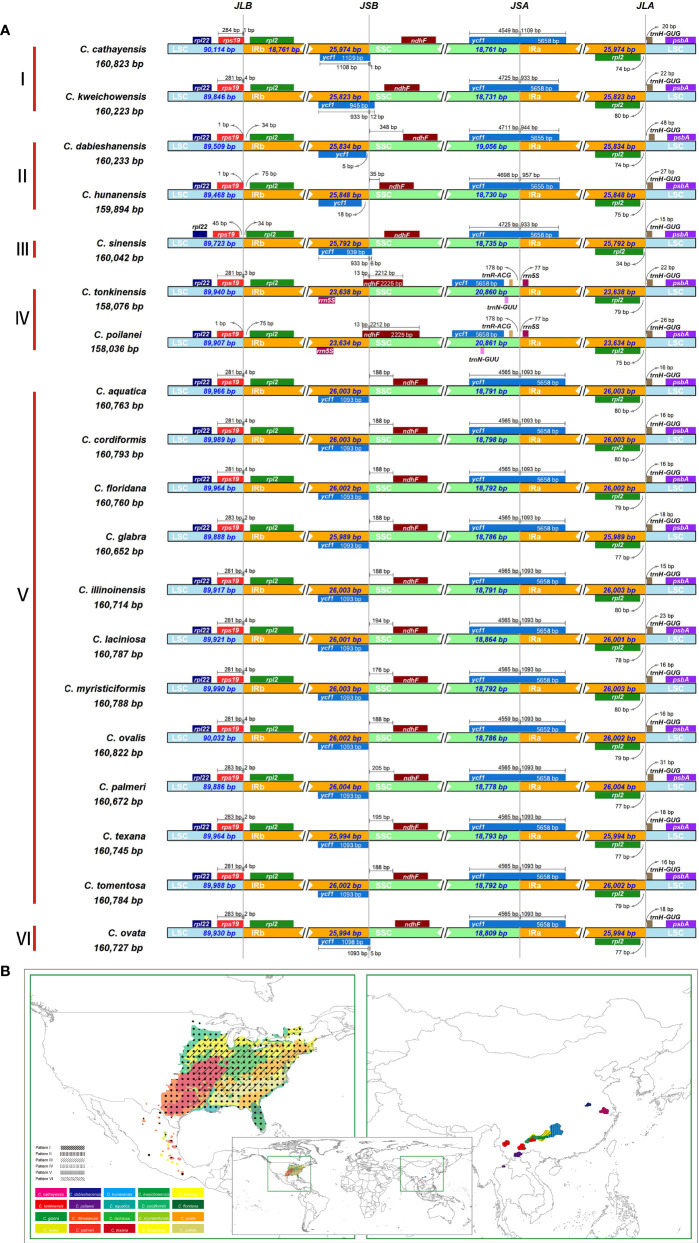
IR junction variation and its diversity among the 19 newly assembled *Carya* plastomes. **(A)**, Comparison of IRb-LSC, LSC-IRa, IRa-SSC, and SSC-IRb boundaries. **(B)**, The modern distribution and the diversity of IR-SC boundaries.

Despite relative conservation of IR/SC boundaries in plant plastomes, contraction and expansion of the IR-SC border regions are common in the process of plastid evolution, which is the major source of variation in angiosperm-plant plastome length (Raubeson et al., 2007; Wang et al., 2018). We examined the fluctuation of IR-SC borders together with the adjacent genes in the 19 *Carya* plastomes. Of the IR-SC junctions, IRa/LSC junction has the most conserved border within a gene spacer of *rpl2* to *trnH-GUG*, with contraction sizes of 34 - 80 bp in the IRa region and 15 - 48 bp in the LSC region in the *Carya* plastomes (**Figure 2A**). Complete comparisons of these junctions revealed six patterns: Patterns I to VI (**Figure 2A**). Among them, Patterns I to IV are present in the 7 EA species and Patterns V and VI are present in the 12 NA species (**Figure 2**).

In details, Pattern I includes two species, *C. cathayensis* and *C. kweichowensis*, in which the LSC/IRb border is located within the *rps19* gene with the expansion of 1 bp and 4 bp from *rps19* of LSC to the IRb region, and the IRb/SSC border is located within the *ycf1* fragment with expansion 1 bp and 12 bp from *ycf1* fragment of IRb region to the SSC region (**Figure 2A**). Pattern II consists of *C. dabieshanensis* and *C. hunanensis*, in which the LSC/IRb border is within the gene spacer of *rps19-rpl2* with 1 bp contraction of *rps19* in the LSC region and 34 or 76 bp contraction of *rpl2* in the IRb region. The IRb/SSC border within the gene spacer of the *ycf1*-fragment and *ndhF* that contracted 5 bp and 14 bp of the *ycf1* fragment in the IRb region and 350 and 32 bp in the SSC region, and their SSC/IRa border was similar to Pattern I (**Figure 2A**). Pattern III includes only *C. sinensis*, which similarly has borders of LSC/IRb and SSC/IRa with the same plastomes as in Pattern II and IRb/SSC border with the same plastomes as in Pattern I. Pattern IV contains *C. poilanei* and *C. tonkinensis* plastomes, with a distinguished IR-SC boundary pattern: IRb/SSC border within the *ndhF* gene with a 6-bp expansion from the SSC region to the IRb region, and a SSC/IRa border located in the gene spacer between *trnR-ACG* and *rrn5S* with contractions of 178 bp and 77 bp for both sides, respectively (**Figure 2A**). Meanwhile, the LSC/IRb border of the plastomes in Pattern IV is similar to those in Pattern II (**Figure 2A**). Of the 12 NA *Carya* species, 11 have the highly conserved SC-IR boundaries and were assigned into Pattern V, which has the LSC/IRb border within *rps19* with 2 bp or 4 bp expansion to the IRb region, the IRb/SSC border on the last base of the *ycf1*-fragment in IRb and SSC/IRa border within the *ycf1* gene with 1,093 bp expansion to IRa region (**Figure 2A**). Pattern VI includes only *C. ovata*, with the same borders of LSC/IR and SSC/IRa, except for the IRb/SSC border, which was within the *ycf1*-fragment with 5-bp expansion to the IRa region (**Figure 2A**). These results indicated more conserved SC-IR boundary patterns among NA *Carya* plastomes than those in EA that exhibit highly diverse IR-SC boundary patterns (**Figure 2B**).

### Simple-sequence repeats among *Carya* plastomes

**Figure 3:**
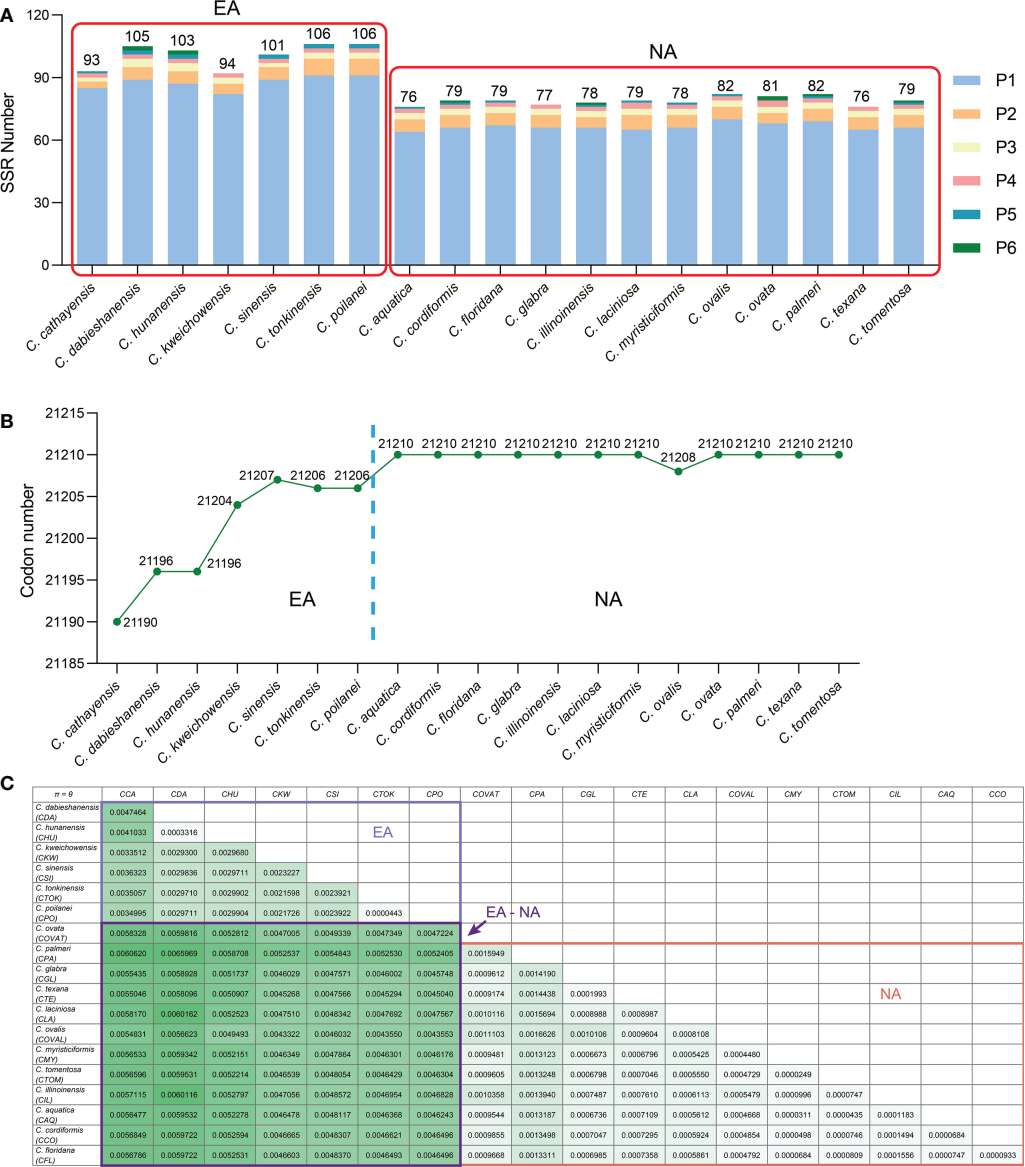
Comparison on SSR numbers, codon number and nucleotide diversities of *Carya* plastomes. **(A)**, Statistics of SSR number for each *Carya* plastome. **(B)**, Statistics of codon number for each *Carya* plastome. **(C)**, The paired nucleotide diversities of *Carya* plastomes from species in Eastern Asia (EA) or North America (NA), or between two species from Eastern Asia and North America (EA-NA).

Simple-sequence repeats (SSRs), also known as microsatellites, are short tandem repeat DNA sequences that consist of repeating 1-6 nucleotide motifs widely distributed throughout the plastomes, which are important molecular markers for analysis of genetic diversity and relationships between species (Yang et al., 2011; Jiao et al., 2012). We detected 1,652 SSRs in the 19 *Carya* plastomes and the numbers of SSRs varied among the species, with a range from 76 in *C. aquatica* and *C. texana* to 106 in *C. poilanei* and *C. tonkinensis* (**Figure 3A**; **Supplementary Table 5**). Of these detected SSRs, mononucleotide repeats were the most abundant SSR motifs, which accounted for approximately 85.5% of the total SSRs, and over 99% of the mononucleotide repeats were composed of A/T repeats (**Figure 3A**; **Supplementary Figure 4**). While the tri- to hexanucleotide SSRs were occupied 7.75% of the total (**Figure 3A**; **Supplementary Figure 4**). In addition, the total number of SSRs in each *Carya* plastome also revealed significantly different patterns among species in EA (93 - 106) and NA (76 - 82) *Carya* species, in which the mononucleotide repeats (especially for A/T repeats) are the major contributor (**Figure 3A**; **Supplementary Table 5**). Meanwhile, the numbers of SSRs of the plastomes can also be classified into six patterns, corresponding to those of IR-SC boundary patterns (**Figures 2**, **3A**).

### Codon usage analysis

Codon distribution analysis of 52 protein-coding genes with a length over 300 bp for each gene sequence in the *Carya* plastomes revealed a similar codon usage pattern among the plastomes of *Carya* species and *J. regia* (Supplementary Table 6). The protein-coding sequences contain a total of 21,190 to 21,210 codons in the *Carya* plastomes and 21,265 in *J. regia*, including stop codons. The total number of codons varied among EA *Carya*, whereas 11 of the NA *Carya* have the same number of total codons (21,210), with the only exception of NA species in *C. ovalis* plastome with 21,208 codons (**Figure 3B**). The total number of codons for each species can also be classified into six patterns as mentioned above.

Other than the codon distribution, we also counted the average effective numbers of codon (ENC) in the plastomes. The results showed that 17 of the 19 assembled *Carya* plastomes had similar codon usage bias (ENC ranged from 48.883 to 49.001), which were slightly lower than that of *J. regia* (48.776) (Supplementary Table 6). *C. tonkinensis* and *C. poilanei* have ENC = 56.51 and 56.52, which are significantly higher than any of other species. All the *Carya* plastomes have similar GC content in the codons (37.32% ~ 37.39%) except for *C. tonkinensis* and *C. poilanei* plastomes which have lower GC contents for the first (39.59%) and second codons (31.12% and 31.18%) and relatively high GC content (41.26% and 41.20%) for the third position (**Supplementary Table 6**). Among all codons in the 19 *Carya* plastomes, leucine is the most abundant amino acid, while cysteine showed the least abundance (**Supplementary Figure 6A**). The most frequent synonymous codon in the *Carya* plastomes was AUU, which encodes isoleucine, and the least frequent codon, except for the stop codons, is UGC, which encodes cysteine (**Supplementary Figures 5, 6B**). Of the 20 amino acids and stop codons, only leucine each has six codon types, while methionine and tryptophan preferred one codon type in the plastomes of 19 *Carya* species and *J. regia* (**Supplementary Figures 6**). In general, AUG encodes not only methionine but also the universal start codon for the nuclear genome of eukaryotes (Thach et al., 1966). We detected that the genes of *ndhD*, *rpl16*, *ycf15*, and *rps19* use ACG, ATC, CTG, and GTG encodes (not ATG) to encode the start codon – AUG respectively.

We also assessed the relative synonymous codon usage (RSCU), a good indicator for measuring nonuniform synonymous codon usage bias in coding sequences (Lee et al., 2010). Both methionine and tryptophan have RSCU = 1 (Supplementary Figure 7), indicating that codons AUG and UGG have no bias or preferences. The plastomes of 19 *Carya* species and *J. regia* had 30 biased codons with RSCU > 1, and their third position is A/U except for leucine (UUG). UUA has the highest RSCU values (1.94 to 2.03) in leucine in all the plastomes of *Carya* species and *J. regia*, and AGC has the lowest RSCU values in leucine (0.38 for *Carya* species and *J. regia*). Moreover, leucine showed A or T (U) bias in all synonymous codons: UUA, UUG, CUU, CUC, CUA, and CUG. We observed that the RSCU value for the specific amino acid increased with the number of codons. The following codons have high RSCU frequency (>30%) and fraction: GAU (aspartate), GAA (glutamate), AUU (isoleucine), AAA (lysine), UUA (leucine), AAU (asparagine), UAU (tyrosine), UUU (phenylalanine), and CAA (glutamine); and the bias toward these nine codons was consistent with the low content of GC in the third codon position (**Supplementary Figure 7**). Our analysis also showed that the RSCU value increased with the quantity of codons coding for a specific amino acid.

### Prediction of RNA editing

We found that 62 post-transcriptional RNAs have editing modifications in 23 protein-coding genes (**Supplementary Figure 9**; **Supplementary Table 7**). Genes of *ndhB* (11 editing sites) and *ndhD* (10 editing sites) contain the most RNA editing sites, followed by *rpoB* and *rpoC2*, with 6 and 5 editing sites respectively. Only one to three RNA editing sites were found in the rest of the 19 protein-coding genes. All of RNA editing sites potentially caused the conversion from cytosine to uracil after transcription, and 43 of them (69.4%) took place at the second nucleotide of codons, with two times the conversion rate compared to the first nucleotide (19, 30.6%) (**Supplementary Figure 8**). However, no correlation was observed between gene length and gene number (**Supplementary Figure 8**). Approximately 77% of the RNA modifications resulted in the conversion of hydrophilic to hydrophobic amino acids, mainly serine to leucine or phenylalanine (S to L, 22 editing sites; S to F, 8 editing sites), or proline to leucine (P to L, 8 editing sites; Supplementary Table 7). We also observed the conversion from proline to serine in three editing sites, representing the changes of amino acids from nonpolar to polar. On the species level, the majority of the RNA-editing sites were shared by all the *Carya* plastomes and only a few were species-specific, for example, CUC to UUU (L to F) was found only in *C. poilanei* at the amino acid position 71, CGG to UGG (R to W) only in *C. sinensis*, CCA to UCA (P to S) only in *C. floridana*, and CUU to UUU (L to F) only in *C. laciniosa* (Supplementary Table 7).

### Analyses of selection and nucleotide diversity

In order to obtain selection patterns in protein-coding genes, the nonsynonymous (*Ka*) and synonymous (*Ks*) substitutions ratios (*Ka/Ks* = *ω*) were determined for 79 unique protein-coding genes with the comparison of the 19 *Carya* plastomes (**Supplementary Figure 9**; **Supplementary Table 8**). Among the protein-coding genes, *ndhA* and *petA* have *Ka/Ks* ratios greater than 1.0 (**Supplementary Figure 10**; **Supplementary Table 8**), indicating the genes were subjected to positive selection. The rest of the 77 protein-coding genes have *Ka/Ks* < 1.0 (**Supplementary Figure 9**; **Supplementary Table 8**), indicating that purification selection happened only during plastome evolution. Specially, seven genes (*atpF*, *ccsA*, *matK*, *rbcL*, *rpoA*, *rps15* and *ycf1*) have the *Ka/Ks* ratios between 0.5 and 1.0 (**Supplementary Figure 9**; **Supplementary Table 8**). These results clearly indicate that protein-coding genes in plastomes of different plant species were subjected to diverse selection pressures.

Two parameters, Pi (*π*) and theta (*θ*), were used for measuring the nucleotide variability among protein-coding genes of the 19 *Carya* plastomes. The value *π*varied among the protein-coding genes with a range from 0 to 0.08495 (average *π* = 0.00326) (Supplementary Table 8). The gene *rpl36* (*π* = 0.08495) was notably variable among the protein-coding genes in the 19 *Carya* plastomes. The gene *ycf1* showed a relatively lower*π* value (0.00493) among the protein-coding genes in the *Carya* plastomes, although it was commonly used as a representative plant DNA barcoding region (Dong et al., 2015). The variation of the other parameter *θ* showed the same patterns as the *π* value among *Carya* plastomes of the 79 protein-coding genes (**Supplementary Table 8**). In comparison, plastomes of EA *Carya* species revealed higher nucleotide diversities (*π* and *θ*) than those in NA *Carya* species (**Figure 3C**).

### Maternal phylogenetic inference within *Carya*

**Figure 4:**
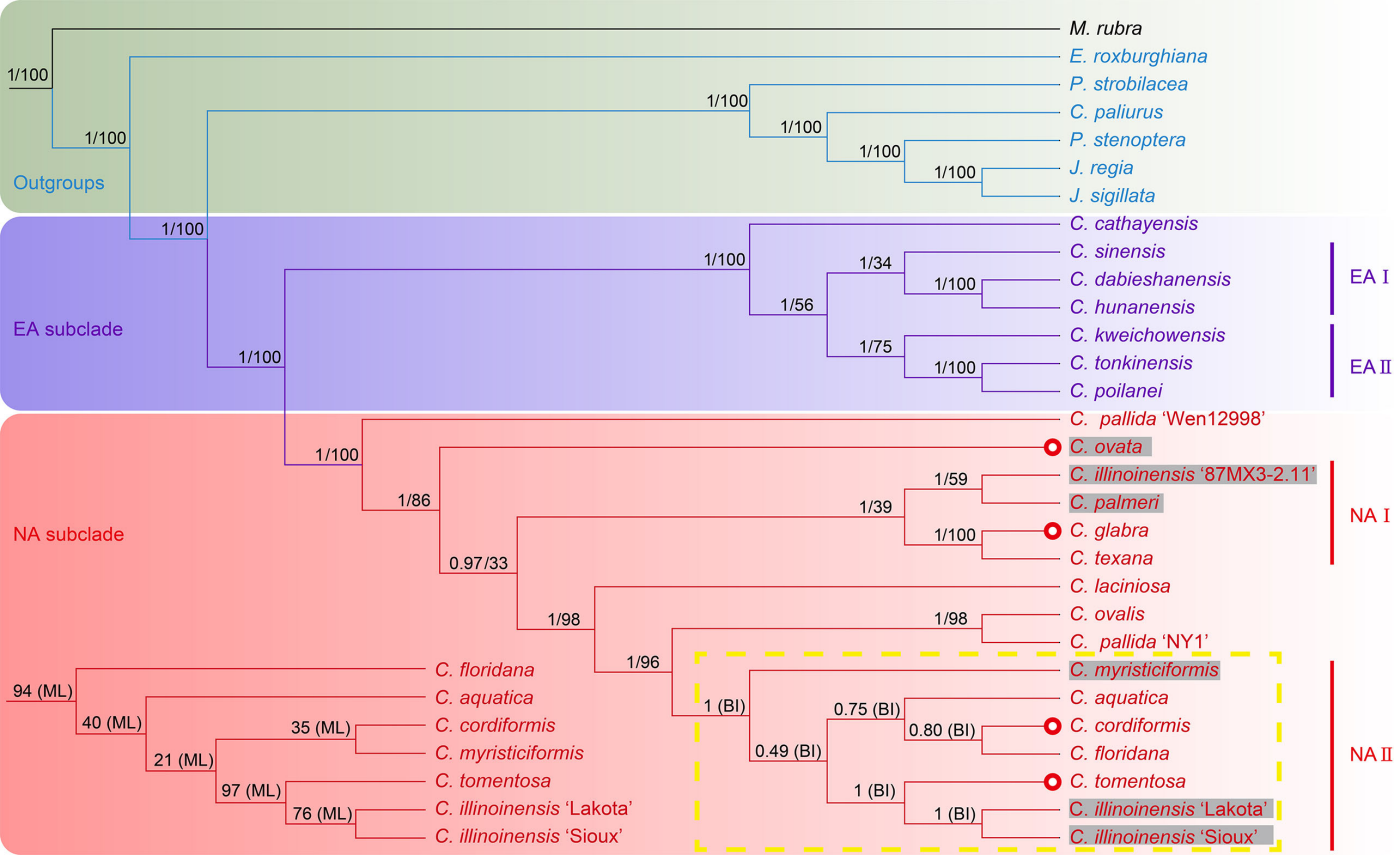
Phylogenetic trees of *Carya* species and representative species in other genera of Juglandaceae inferred from the datasets of complete plastomes using Bayesian inference and maximum likelihood method. The posterior probability (PP) and bootstrap (BS) values that supported each node are shown above the branches. The colors in green, light purple and pink indicate the outgroups, EA and NA *Carya* species, respectively. The letter n represents the haplotype, and x represent and the number before it shows the chromosomal set of the haplotype of each species. The numbers after n and x are the chromosomes of haplotype of each species. Red circles show the species in section *Apocarya*, gray background and the red circles indicate the tropical distributed species/varieties. The lower left quarter and the yellow box respectively show the parts with different topologies in the trees based on ML and BI methods.

The phylogenetic analyses, based on matrices (with indels) from seven datasets containing the full or partial sequence of 30 plastome sequences including 26 Juglandaceae species and one *M. rubra* (as tree root), resulted in seven different topologies using both Bayesian inference and the ML method (**Figure 4**; **Supplementary Figure 10–15**). Among the seven BI trees, one generated from the IR-region dataset displayed a very chaotic phylogenetic relationship among all analyzed species and varieties, and therefore was discarded for further analysis (**Supplementary Figure 15**). The remaining six BI trees displayed identical topologies (with posterior probability (PP) value = 1) on the intergeneric level in Juglandiodeae. All six topologies supported the two sister subclades of EA and NA in the genus *Carya* (**Figure 4**; **Supplementary Figures 10–14**). When considering the overall supporting rates for the six topologies based on BI, we are confident that the best phylogenetic tree is generated by full-length plastome sequence, followed by LSC, LSC-SSC, SSC, CDS, and LSC-IR-SSC (**Figure 4**; **Supplementary Figures 10–14**). Almost all branches of the BI tree inferred from full-length plastomes displayed the highest supports of any of the other 5 phylogenetic trees based on Bayesian inference. The topologies of all ML trees highly supported the backbone relationships among genera of Juglandoideae and the two sister subclades of EA and NA in *Carya* (**Figure 4**; **Supplementary Figures 10–15**). The overall supporting rates were in the same sequence as mentioned above for BI trees (**Figure 4**; **Supplementary Figures 10–15**). Excluding the trees based on the IR dataset, the genus *Engelhardia* formed a sister relationship with five other genera (including *Carya*) with full supports (PP = 1 or BS = 100) in the remaining partial or full-length plastome sequences-based topologies generated by Bayesian inference and the ML method (**Figure 4**; **Supplementary Figures 10–14**). These topologies fully supported the hierarchical clustering of *Platycarya*, *Cyclocarya*, *Pterocarya*, and *Juglans*, and grouped a sister clade with *Carya*. Within the *Carya* monophyletic group, two major subclades, i.e., EA and NA are well-separated (**Figure 4**; **Supplementary Figures 10–14**). However, the supporting rates [bootstrap (BS) values] for the branches in all seven ML trees were significantly lower than the PP values in BI trees, and the topologies in the EA and NA subclades varied among the ML trees (**Figure 4**; **Supplementary Figures 10–15**). Compared to the corresponding BI tree, similar phylogenetic topologies were obtained using the ML method based on the data set generated by full-length plastome sequences, which also represented the best ML tree although with relatively low supports (BS values) of most branches (**Figure 4**; **Supplementary Figures 10–15**). Therefore, the BI trees are considered the primary references in the following analyses of plastome phylogenetic relationships of *Carya*, and the detailed analyses were mainly based on the BI topology inferred by full-length plastome sequences (**Figure 4**; **Supplementary Figures 10–14**).

The plastome phylogenetic relationships inferred from datasets of full-length, LSC, and LSC-SSC regions of plastomes displayed the same topologies for EA subclade with PP values of 0.997 (LSC BI phylogenetic tree) to 1 (full-length and LSC-SSC BI trees) (**Figure 4**; **Supplementary Figure 10**). However, the maternal phylogenetic incongruencies were obtained between species of EA or NA *Carya* in the topologies inferred from partial or full-length plastome datasets. In contrast, the maternal phylogenetic trees inferred from the datasets of SSC, CDS, and LSC-IR-SSC showed completely different topologies for EA *Carya* subclades, with relatively low PP values for several internal subclades (**Supplementary Figures 11–13**). However, all six BI topologies support the sister relationships for two pair of terminal branches, *C. dabieshanensis* – *C. hunanensis* and *C. tonkinensis – C. poilanei*, with supports = 1 (**Figure 4**; **Supplementary Figures 11–13**). Within the EA subclade, *C. cathayensis* was considered the base taxon with PP = 1 in four topologies of full-length, LSC-SSC, SSC and LSC-IR-SSC. Meanwhile, the topologies of full-length and LSC fully supported the sister relationships with the nodes of *C. sinensis* – *C. hunanensis – C. dabieshanensis* (designated as EA-I) and *C. kweichowensis* – *C. tonkinensis – C. poilanei* (designated as EA-II) (**Figure 4**; **Supplementary Figures 10–13**). *C. kweichowensis* formed a sister node with the terminal branch of *C. tonkinensis – C. poilanei* in four topologies of full-length, LSC, LSC-SSC, and SSC of plastomes. *C. sinensis* formed a sister relationship with the terminal branch of *C. dabieshanensis* – *C. hunanensis* in the phylogenetic trees of full-length plastomes and LSC-SSC sequences. This relationship was strongly supported in the LSC dataset-based maternal phylogeny but formed a terminal sister relationship with *C. kweichowensis* in the SSC dataset-based phylogenetic tree. Although *C. dabieshanensis* and *C. cathayensis*, have close geographical distribution and similar morphological traits, the former has larger size of fruits and nuts than the latter, and their maternal phylogenetic relationship is relatively far away (**Figure 4**; **Supplementary Figure 17**).

In the NA subclade, five out of six BI topologies (except for that inferred from SSC dataset) fully supported the *C. pallida* var. ‘Wen 12998’ as the basal taxon, while *C. pallida* variety ‘NY1’ formed a terminal branch with *C. ovalis* (**Figure 4**; **Supplementary Figures 10–13**). All six BI topologies fully supported the terminal branch of *C. glabra* and *C. texana*. The variety of *C. illinoinensis* ‘87MX3-2.11’ displayed a sister relationship with *C. palmeri* in the full-length and SSC-based phylogenetic trees, and formed a sister relationship with *C. laciniosa* in the LSC, LSC-SSC, CDS, and LSC-IR-SSC-based phylogenetic trees. The other two varieties of *C. illinoinensis* – ‘Lakota’ and ‘Sioux’ formed a terminal branch with very strong supports and a sister branch to *C. tomentosa* in the full-length, LSC, LSC-SSC, and LSC-IR-SSC-based maternal phylogenetic trees. *C. aquatica* and the terminal branch of *C. cordiformis – C. floridana* formed a sister note in full-length and LSC plastome sequences-based maternal phylogenetic trees. The phylogenetic relationship of *C. ovata* varied in five phylogenetic trees (full-length plastomes, LSC, LSC-SSC, SSC, and CDS-based inferences).

In summary, the maternal phylogenetic relationships within the subclades EA or NA were clear based on the topology inference of complete plastomes with high confidence (PP > 0.8) for most nodes and the topology was selected for further discussion on the maternal phylogenetic relationships within EA or NA subclade (**Figure 4**). The topology of the EA subclade fully supported the earliest divergence of *C. cathayensis*, which consists of a sister relationship with two sub-sister nodes, *C. sinensis* – *C. hunanensis – C. dabieshanensis* (EA-I) and *C. kweichowensis* – *C. tonkinensis – C. poilanei* (EA-II), indicating that they came from a common ancestor in the genus. Among the species of the NA subclade, one landrace ‘Wen 12998’ of *C. pallida* was clustered as a basal taxon and formed a sister relationship with the rest of the NA plastomes. Except for the node in NA-II (PP = 0.502) containing *C. tomentosa*, two cultivars of *C. illinoinensis*, and the sister node (PP = 0.755) of *C. aquatica*, *C. cordiformis* and *C. floridana*, the majority nodes of the NA subclade topology displayed very high supporting rates. The topology of NA subclade disrupted the morphological features-based division of the two sections, *Carya* and *Apocarya* (Huang et al., 2019), but varieties that are at the same terminal branch or that have a close relationship in the phylogenetic tree indicate that they have close or overlapping distribution regions (**Figures 2B**, **4**; **Supplementary Figure 17**). Two pairs of terminal branches containing a local collection ‘87MX3-2.11’ of *C. illinoinensis* (Wang et al., 2020), and *C. palmeri* (section *Carya*) section and species *C. glabra* and *C. texana* (section *Apocarya*) consisted of an internal sister node (NA-I) with *C. ovata* (section *Carya*) in the NA subclade (**Figure 4**). In contrast, *C. myristiciformis*, as the partial sister of the terminal branch of *C. ovalis* and the *C. pallida* var. ‘NY1’, showed a relatively distant maternal phylogenetic relationship with the two species (**Figure 4**). Interestingly, two varieties of *C. illinoinensis* – ‘Sioux’ and ‘Lakota’ which had different parentages and came from different controlled crosses made in Brownwood, Texas in 1943 and 1964, respectively (Grauke and Thompson, 1996; Wang et al., 2020), also showed distant phylogenetic relationships with the wild seedling ‘87MX3-2.11’ that originated from Oaxaca, Mexico (Wang et al., 2020) (**Figure 4**).

## Discussion

### Characteristics and comparison of *Carya* plastomes: Conservation and diversity

Being consistent with reports in most flowering plants, *Carya* plastomes have conservative canonical quadripartite structures including LSC and SSC regions (**Figure 1**), which are separated by two IR regions that are generally known to play a role in the structural stability of plastomes (Palmer and Thompson, 1982; Bock, 2007). The *Carya* plastomes are similar in gene content and gene order (113 unique genes) (**Figure 1**; **Tables 1**, **2**), as reported in many angiosperms (Hu et al., 2016; Xu et al., 2017; Mader et al., 2018; Yang et al., 2018). A strong tendency toward A or U at the third codon position (Clegg et al., 1994; Gao et al., 2017; Mader et al., 2018; Meng et al., 2018) may explain why the A/T content is as high as ~ 64% in *Carya* plastomes (**Table 1**). The conservatism of *Carya* plastomes is also revealed by RNA editing sites and the measurement of selection for each protein-coding gene (**Supplementary Table 7**).

As the most conserved region in the plastomes, IR expansion or contraction may alter the plastome size and the stability of the genomic structure, which in turn could cause IR-SC boundary shift, repeated sequences (Dugas et al., 2015), and/or duplication or deletion of a certain gene through inversion during evolution (Park et al., 2018). Frequent expansions and contractions at the junctions of IR-SCs have been recognized as evolutionary signals for illustrating the relationships among taxa (Khakhlova and Bock, 2006; Raubeson et al., 2007; Wang et al., 2008; Lu et al., 2018; Park et al., 2018). Moreover, repeated sequences, especially SSRs play key roles in plastid genome rearrangement, divergence, and evolution (Weng et al., 2014). Our analyses reveal the intercontinental disjunctive distribution between and among EA and NA *Carya* species, reflected by the patterns of IR-SC junctions, the number of SSRs and codons, and the paired nucleotide diversities of *Carya* plastomes (**Figures 2**, **3**). The diversified features described here for *Carya* plastomes and the shortened IR regions in *C. tonkinensis* and *C. poilanei* could be related to the variation in genome size and the long evolutionary history of *Carya* species. By comparison, *Carya* species in EA exhibited higher diversities in plastome features and structure compared to those in NA (**Figures 1**–**3**; **Table 1**), although higher diversities in species and morphology were displayed in NA *Carya* than those in EA (Huang et al., 2019). The interesting findings combined with geology and vicariance events suggest more diverse matrilineal sources of EA *Carya* species, in contrast to fewer matrilineal sources of NA *Carya* species during their evolution. Meanwhile, the changes in climates and habitats especially in the glacial period may speed up the adaptive speciation in NA *Carya* and lead to higher speciation and morphological diversities in NA than those in EA. It is known that climate cooling is commonly accepted as the main causes of the isolated habitats and the disjunctions of floristic elements between continents (Raven, 1972; Tiffney, 1985; Wen, 1999). One example as addressed by Deng et al. (2017), is the modern Asia-North America disjunct distribution of two taxa from Rubiaceae – *Kelloggia* and *Theligonum* formed by the fragmentations of original wide distribution caused by climate cooling. Extinction of *Carya* in EA and NA might occur commonly during climate change. The rapid uplift of Qinghai-Tibetan Plateau and climate change could be responsible for the modern restricted distribution of *Carya* in EA (**Supplementary Figure 16**), while the overlapped distribution area and frequent inter- and intra-species hybridization could account for the relatively low plastome diversity in modern NA *Carya* species (**Supplementary Figure 16**).

### Complete plastomes: An excellent tool for inferring the matriarchal phylogeny and geography of *Carya*

Our maternal phylogenetic analyses showed that six of the BI-based and all ML-based topologies inferred by different datasets of plastomes support the sister relationship of *Carya* and the monophyletic *Cyclocarya*-*Juglans*-*Platycary*-*Pterocarya* clade in Juglandoideae (**Figure 4**; **Supplementary Figures 10–13**). Our results also strongly support two monophyletic subclades corresponding to the disjunctive geographical distributions of *Carya* in EA and NA (**Figure 4**; **Supplementary Figures 10–13**). The results are highly consistent with those of previous inferences on the intergeneric phylogenetic relationships in Juglandoideae and the relationships between two subclades in the genus *Carya*, based on the molecular markers from partial or complete nuclear or/and organelle sequences (Manos and Stone, 2001; Manos et al., 2007; Zhang et al., 2013; Zhang et al., 2019; Mu et al., 2020; Zhang et al., 2021; Zhou et al., 2021). It is known that the plastome is a key organelle in the plant cells, performing photosynthesis and other metabolic processes related to the adaptation of the plant to its environments (Dierckxsens et al., 2016). Although the basic structure of the plastome is highly conserved throughout land plant lineages, it has been proven that differences in the sizes of the complete genome and the protein-coding gene content of the different genome regions related to the difference in selection pressure are informative in phylogeny and evolution for many plant lineages. Although the matriarchal phylogenetic relationships among species and/or varieties within the EA and NA subclades varied among the topologies inferred from different datasets of plastomes, the plastome-based physiologies still provide very important clues to the backbone relationships between EA and NA *Carya* (**Figure 4**; **Supplementary Figures 10–13**). The plastome-based phylogeny of *Carya* could also provide a good example for exploring the evolution of plastomes as we present in this study.

By comparing the PP and BI values of all nodes among six topologies, almost all the nodes of the BI tree reconstructed from full-length plastomes showed the highest support (PP > 0.8) (**Figure 4**; **Supplementary Figures 10–13**). Therefore, the full-length plastome dataset-based BI tree is considered the main reference for further analyses of maternal phylogenetic relationships among *Carya* species. The maternal phylogenetic relationships among EA *Carya* species are highly in agreement with their geographical distributions (**Figures 2B** and **4**; **Supplementary Figure 17**). The maternal phylogenetic positions of the species in NA subclade provide strong support for their overlapping and adjacent distribution patterns (**Figures 2B** and **4**; **Supplementary Figure 17**), although the topology does not match the morphological features-based division of two sections (Manos and Stone, 2001; Huang et al., 2019). Meanwhile, the topologies among species within EA and NA subclades exhibited significant deviation from the pattern presented by nuclear genome data (Huang et al., 2019). Our results demonstrated that the different contributions of nuclear and organelle genomes, compared to the control, based on the morphological features associated with the different evolutionary rates between organelle and nuclear genomes, showed the difference between topologies based on the different datasets of plastomes in the present study. In general, the nonparental-inherited plastomes are highly conservative in protein-coding gene functions and genome structure, which can also reveal the maternal origin and diversity of a plant (Raubeson and Jansen, 2005). However, the morphological characteristics could be controlled by parental-inherited materials in the nucleus and may be influenced by environment and selection resulting in adaptation, convergent evolution, and species diversity (Givnish, 2016). Taking this into account, it is easy to understand the contradiction between topologies built from plastomes and nuclear data, as both had different evolutionary trajectories.

*Carya poilanei*, has been regarded as an extinct species in the subfamily Juglandoideae for more than 60 years before its rediscovery in the Ailao Mountain area, Jianshui County, Yunnan Province, China (Zhang et al., 2022). This species has historically been classified as a member of Juglans (Chevalier, 1941), then moved to the genus *Carya* (Leroy, 1950), and finally botanically characterized (Leroy, 1955). Although it is classified in the section *Sinocarya* of the genus *Carya* based on its morphological traits, the phylogenetic and maternal relationships are still unclear. The rediscovery of the species brings this issue back into consideration. Our results indicate that *C. poilanei* has the highest similarity with *C. tonkinensis* in plastome structure and features, and it has the closest maternal phylogenetic relationship with *C. tonkinensis*, which is supported by their closest geographical distribution (**Figures 1**–**4**; **Tables 1**, **2**; **Supplementary 17**). Therefore, we assume that these two species historically shared a common maternal ancestor. In addition, the taxonomic placement of four contentious species *C. dabieshanensis*, *C.* (*Annamocarya*) *sinensis*, *C. glabra*, and *C. ovalis* has been disputed for decades (Chang and Lu, 1979; Thompson and Grauke, 1991; Manos and Stone, 2001; Grauke and Mendoza-Herrera, 2012; Grauke et al., 2016). *C. dabieshanensis* has been treated as a member of *C. cathayensis* because of their high similarities in morphological features, except for the larger fruit size of *C. dabieshanensis* (Liu and Li, 1984). Our previous nuclear genome analyses also supported that *C. dabieshanensis* is close to *C. cathayensis*, but not to any members in the section Sinocarya (Huang et al., 2019). This study provided a full comparison of the morphological features between these two species and found no additional differences beyond the genomes (**Supplementary Figure 17**). However, the plastome features of both species reveal a significant variation in the patterns of IR-SC junction: *C. cathayensis* belongs to Pattern I with *C. kweichowensis*, but *C. dabieshanensis* belongs to Pattern II with *C. hunanensis* (**Figure 2A**). The phylogenetic topology built from the complete plastomes also supports that *C. dabieshanensis* has the close matrilineal relationship to C. hunanensis but not to *C. cathayensis* (**Figure 4**). Although taxonomists distinguished *C.* (*Annamocarya*) *sinensis* from the genus *Carya* by its distinctive taxonomic, botanical, and horticultural characteristics, high throughout genome-wide sequencing technology has provided a credible molecular phylogeny to distinguish plant species (Favre et al., 2020). The plastomes-based phylogenetic tree here also provided solid support that *C.* (*Annamocarya*) *sinensis* is a member of EA *Carya* (**Figure 4**). From the plastome phylogenetic tree, *C. glabra* and *C. ovalis* were separated in the NA *Carya* subclade and this is also confirmed by their plastome features, the patterns of IR-SC junction variations (**Figures 2A**, **4**), as well as their morphological differences (Grauke, 2003). As such, the matriarchal-originating plastomes provide an effective tool for taxonomic placement of the outcrossing plant species, at least at genus level.

### New insight into the inconsistency between phylogenetic topologies of *Carya*

As mentioned above, maternal phylogenetic analyses based on whole plastomes and partial plastome regions, except for the IR regions, recovered the same topologies and provided substantial support of the six genera of Juglandoideae and two subclades of the genus *Carya* (**Figure 4**; **Supplementary Figures 10-16**). These results were in consistent with our early studies based on nuclear genome data and a few organelle and nuclear gene markers (Zhang et al., 2013; Huang et al., 2019). However, multiple significant inconsistencies between species of EA or NA subclades have been found in the maternal phylogenetic topologies inferred from different plastome regions and the nuclear datasets. These discordances of phylogenetic trees have been reported in numerous studies of plants in the North Hemisphere, including several close relative families of Juglandaceae, such as Betulaceae and Fagaceae in Fagales (Chan and Ragan, 2013; Lemmon and Lemmon, 2013; Zwickl et al., 2014; Stenz et al., 2015; Yang et al., 2019; Yang et al., 2021; Zhou et al., 2022). The inconsistencIES between the phylogenetic relationships based on plastomes and nuclear genes are believed to be based upon hybridization and introgression, incomplete lineage sorting, and/or ancient chloroplast capture, while the gene flow caused by hybridization and introgression, as well as plastid capture between heterocompatible species, is considered to be the source of phylogenetic inconsistencies inferred from different datasets of plastomes (Suh et al., 2015; Yang et al., 2019; Lovell et al., 2021; Yang et al., 2021; Zhou et al., 2022).

Hedrick (2013) found that introgression usually involves a small number of genes or genomic regions but may be of substantial significance. Incomplete lineage sorting largely resulted from ancestral polymorphism spanning multiple speciation events and subsequent random fixation of the polymorphisms in different lineages (Oliver, 2013), and it is common in multi-locus phylogenetic datasets due to rapid diversification (Wang et al., 2022). The entire plastome functions as a single linked locus, with different haplotypes retained through ancient clado-genic events like the alleles of a single nuclear locus (Folk et al., 2017; Lee-Yaw et al., 2019). Natural hybridization and introgression are very common in numerous wind-pollinated plant species, such as Betulaceae (Yang et al., 2019). In such species, plastid DNA is maternally inherited from ovum, whereas nuclear DNA is transmitted parentally through both pollen and ovum. Ancient and recent hybridization and introgression can result in rapid introgression of maternally inherited plastomes and the concerted evolution of the nuclear genes toward the introgressive species, especially for the geographically closely related species, and finally distort the phylogenetic relationships of introgressive species. In *Carya*, hybridization has been well studied and introgression signatures between species were evident in previous and recent reports (Lovell et al., 2021; Wang et al., 2022). In addition to the incomplete lineage sorting and ancient plastid capture, the obvious incongruencies within subclades of *Carya* between plastome- and nuclear marker-based topologies in our analysis could partially result from introgressive hybridization. Introgressive hybridization could also account for the phylogenetic incongruencies between varieties of non-monopoly *Carya* species such as *C. illinoinensis* and *C. pallida*.

‘87MX3-2.11’ (*C. illinoinensis*), a local seedling collection from an autochthonous tree growing near Zaachilla, Oaxaca in Mexico (Wang et al., 2020), was clustered with *C. palmeri*, which is only distributed in the tropical region in the NA *Carya* subclade (**Figures 2B**, **4**; **Supplementary Figure 16**). Meanwhile, two controlled-cross cultivars of *C. illinoinensis*, ‘Lakota’ and ‘Sioux’, made in Brownwood, Texas, in 1964 and 1943, respectively (Grauke and Thompson, 1996) were grouped together (**Figure 4**). ‘Schley’ is the maternal parent of ‘Sioux’, while ‘Lakota’ was from a cross of ‘Mahan’ × ‘Major’, and ‘Mahan’ is a progeny of ‘Schley’ (Thompson and Young, 1985; Grauke et al., 2015). Therefore, it is easy to understand the sister relationships between these two cultivars (**Figure 4**). Meanwhile, the sister group of ‘Lakota’ and ‘Sioux’ demonstrates a close maternal phylogenetic relationship with the widely distributed species *C. tomentosa* (**Figures 2B**, **4**; **Supplementary Figure 17**). Our plastome-based phylogenetic analyses also suggested that it is likely that the *C. illinoinensis* ‘87MXx3-2.11’ share a common matrilineal ancestor with the tropical-originated *C. palmeri*, and ‘Lakota’ and ‘Sioux’ share a common female ancestor with *C. tomentosa* because of their overlapping and/or adjacent distribution (**Figure 2B**; **Supplementary Figure 17**). In addition, two *C. pallida* landraces ‘Wen 12998’ and ‘NY1’ collected from different locations of the distribution have close maternal phylogenetic relationships with *C. ovata* and *C. ovalis*, respectively. This pattern also supports the model of “the maternal phylogenetic relationship of intra-genus outcrossing species is determined by geographical distribution, and the geographically adjacent species shares a common maternal ancestor adjacent geographical distribution sharing common maternal ancestor” like the relationships among the cultivars in *C. illinoinensis* (**Figure 4**). Different varieties within the same species clustered in different phylogenetic positions may be caused by their multiple matrilineal origins, for reasons such as overlapping and/or adjacent distribution regions, the hybridizability of interspecies of *Carya* species, or diverse plastome structure features, or wind pollination (Wang et al., 2022). These results may partially account for the phylogenetic incongruencies between species in EA or NA subclades inferred from partial or complete plastome datasets or nuclear data. The results generated here indicate that further broader population-wide sampling and their plastome assemblies will be a powerful approach in tracking the matrilineal historical origin of a species, especially for outcrossing species.

## Data availability statement

The datasets presented in this study can be found in online repositories. The names of the repository/repositories and accession number(s) can be found below: https://www.ncbi.nlm.nih.gov/genbank/, MW410235 MW410236 MW368388 ON568300 MW410227 MW255965 MW368387 MW410228 MW410229 MW410230 MW298527 MW410238 MW410237 MW410231 MW410232 MW440674 MW410233 MW410234 MW421595.

## Author contributions

LX: Conceptualization. JX, SL, WZ and JW: Investigation, Resources. GX: Resources. JX and SL: Validation. HG, JH and YY: Data Curation. LX, JX, JZ and KW: Data Curation, Visualization. LX, JX and WZ: Writing Original Draft. LX, JX, WZ and XW: Writing, Review and Editing. LX, JZ: Supervision. All authors contributed to the article and approved the submitted version.

## Funding

This work was supported by grants from the Chinese Ministry of Science and Technology, China (grant no. 2018YFD1000604), the Natural Science Foundation of Zhejiang Province, China (grant no. Z20C160001), Research and Development Fund of Zhejiang A&F University (grant no. 2018FR002) and the State Key Laboratory of Subtropical Silviculture in Zhejiang A&F University, China (grants no. ZY20180202 and KF201905).

## Acknowledgments

We are grateful to Dr. Zhiduan Chen at Institute of Botany, the Chinese Academy of Sciences, China, for his help and valuable suggestions during the manuscript preparation. We also thank to Drs. Dayong Zhang and Weining Bai at State Key Laboratory of Earth Surface Processes and Resource Ecology and Ministry of Education Key Laboratory for Biodiversity Science and Ecological Engineering, College of Life Sciences, Beijing Normal University, Beijing, China, for the sharing the resequencing data of *C. poilanei*.

## Conflict of interest

The authors declare that the research was conducted in the absence of any commercial or financial relationships that could be construed as a potential conflict of interest.

## Publisher’s note

All claims expressed in this article are solely those of the authors and do not necessarily represent those of their affiliated organizations, or those of the publisher, the editors and the reviewers. Any product that may be evaluated in this article, or claim that may be made by its manufacturer, is not guaranteed or endorsed by the publisher.
