# Supplementary material for: Comparative plastomes of Carya species provide new insights into the plastomes evolution and maternal phylogeny of the genus
Source: Front Plant Sci. 2022 Oct 13;13:990064. doi: 10.3389/fpls.2022.990064 (PMC9667483; doi:10.3389/fpls.2022.990064)
Supplement: Supplementary file 1 [file DataSheet_1.pdf]

Supplementary Figures and Tables

Supplementary Figures and Legends

Supplementary Figure 1. Sampled tree and the habitats of *C. poilanei*.

(A)

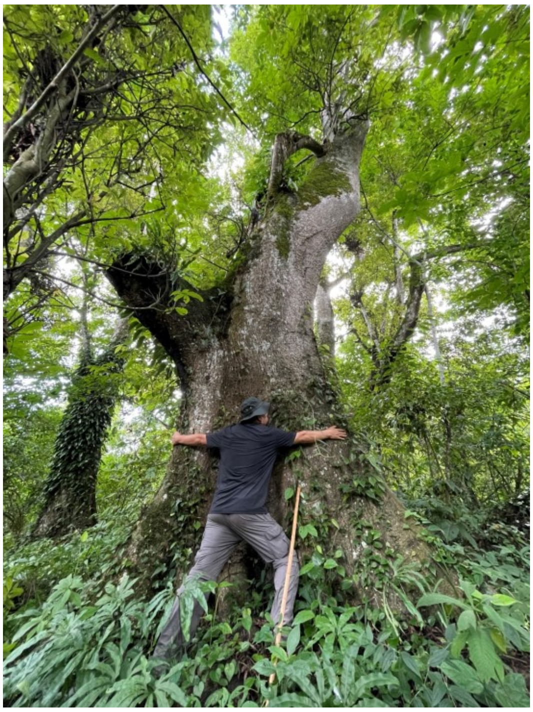

(B)

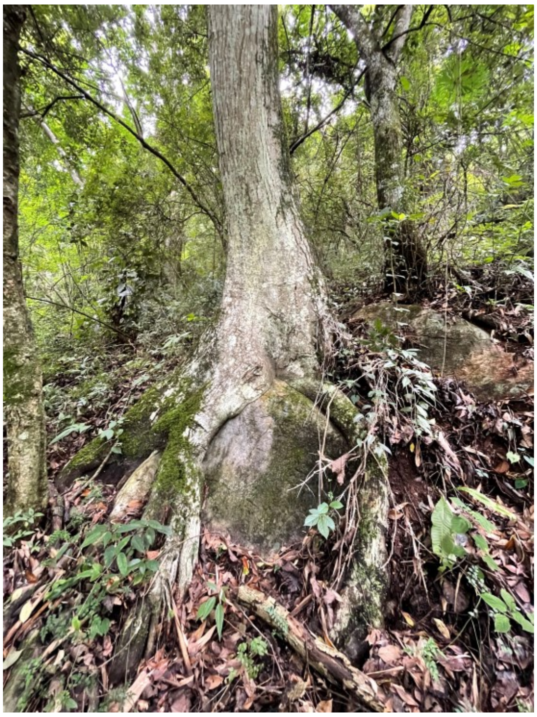

(C)

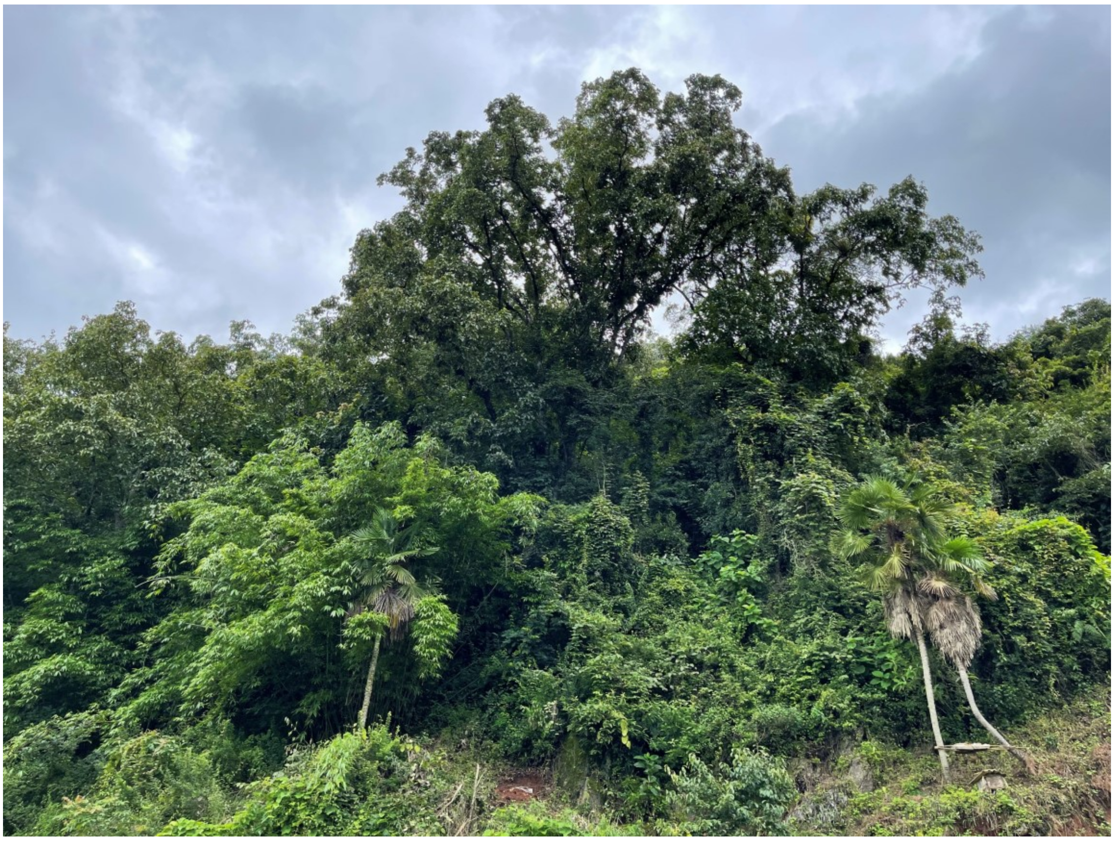

Supplementary Figure 2. The map of each newly assembled *Carya* plastome.

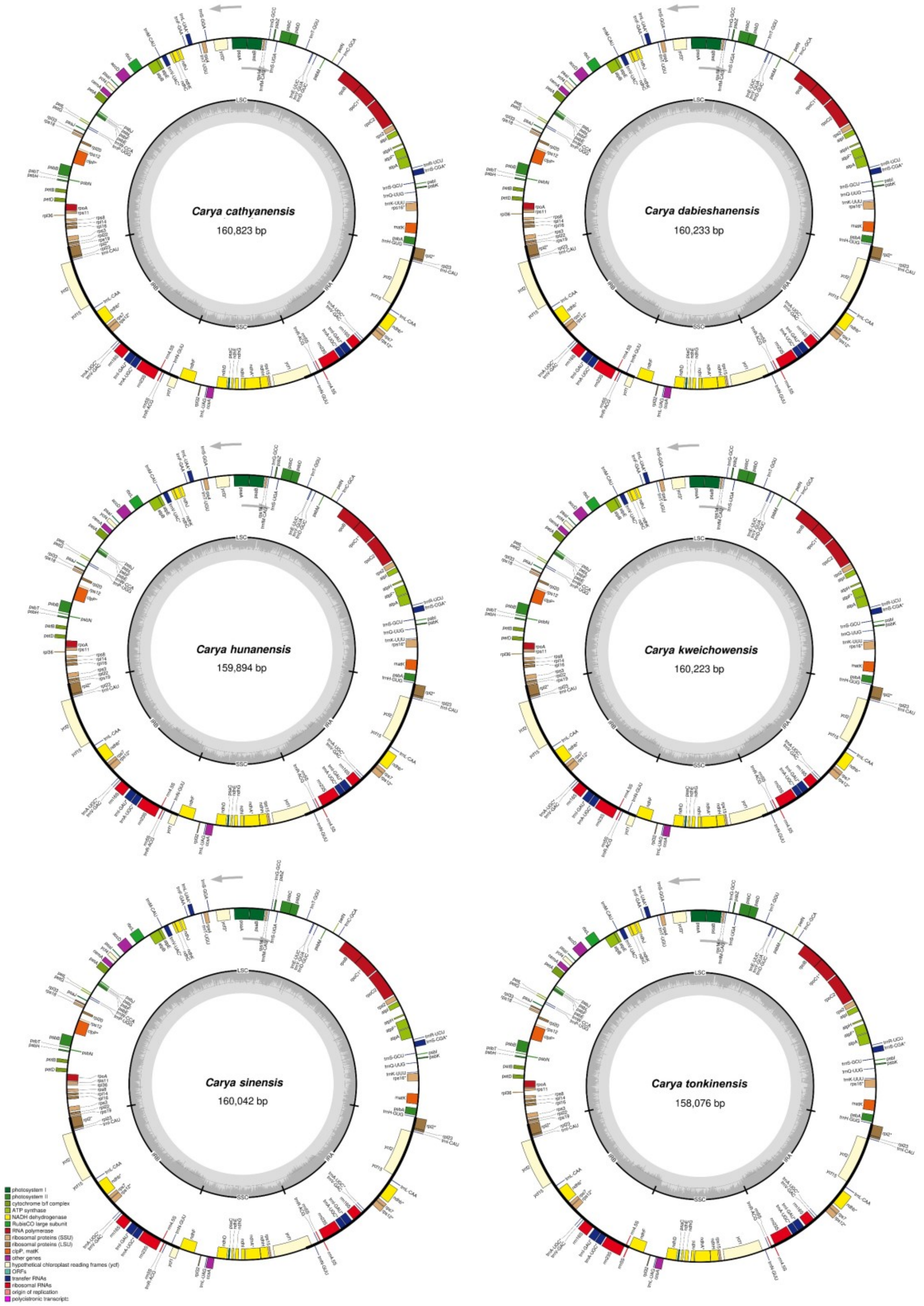

Continued.

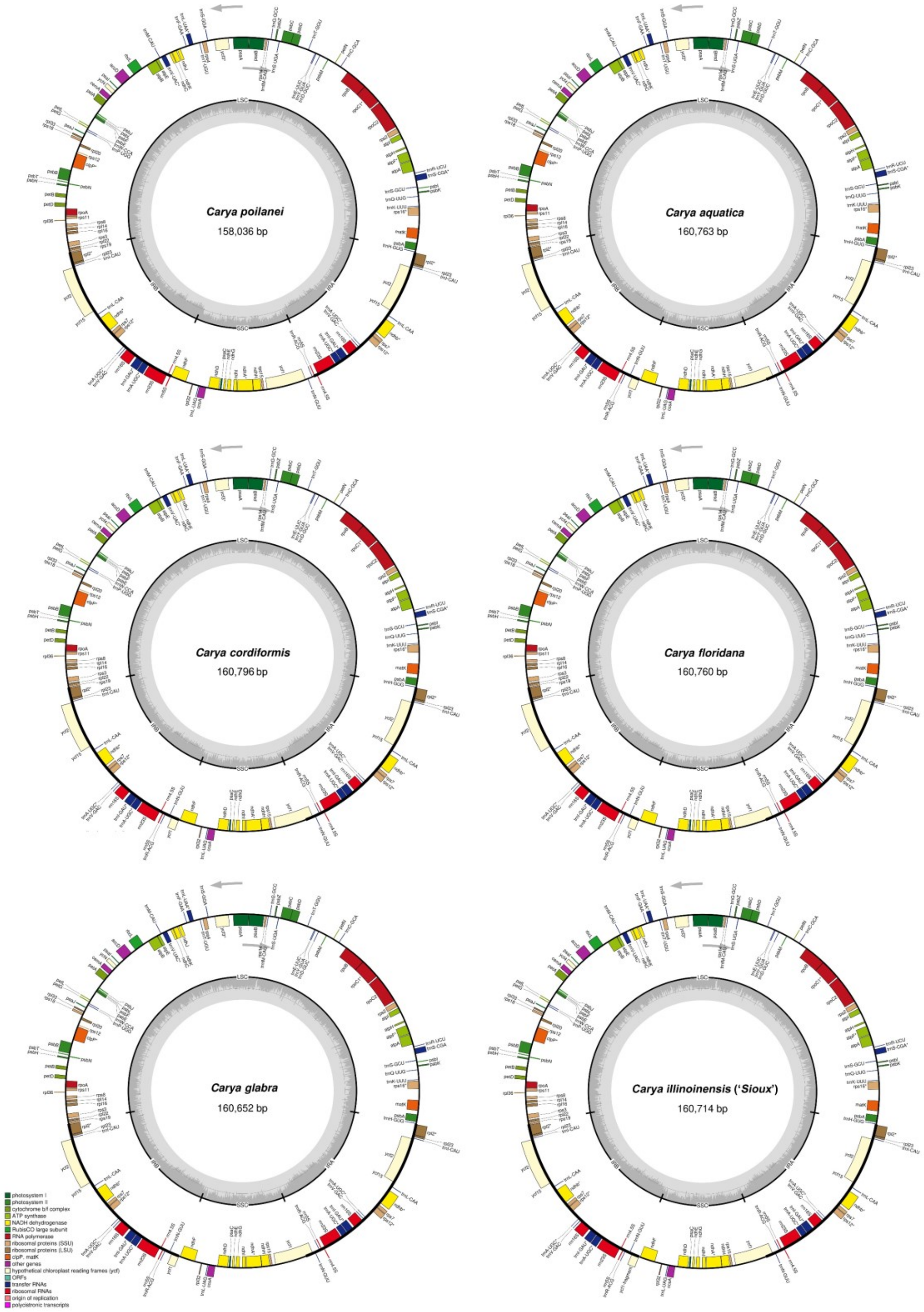
Carya poilanei
158,036 bp
Carya aquatica
160,763 bp
Carya cordiformis
160,796 bp
Carya floridana
160,760 bp
Carya glabra
160,652 bp
Carya illinoinensis ('Sioux')
160,714 bp
photosystem I
photosystem II
cytochrome b/f complex
ATP synthase
NADH dehydrogenase
RubisCO large subunit
RNA polymerase
ribosomal proteins (SSU)
ribosomal proteins (LSU)
clpP, matK
other genes
hypothetical chloroplast reading frames (ycf)
ORFs
transfer RNAs
ribosomal RNAs
origin of replication
polycistronic transcripts

Continued.

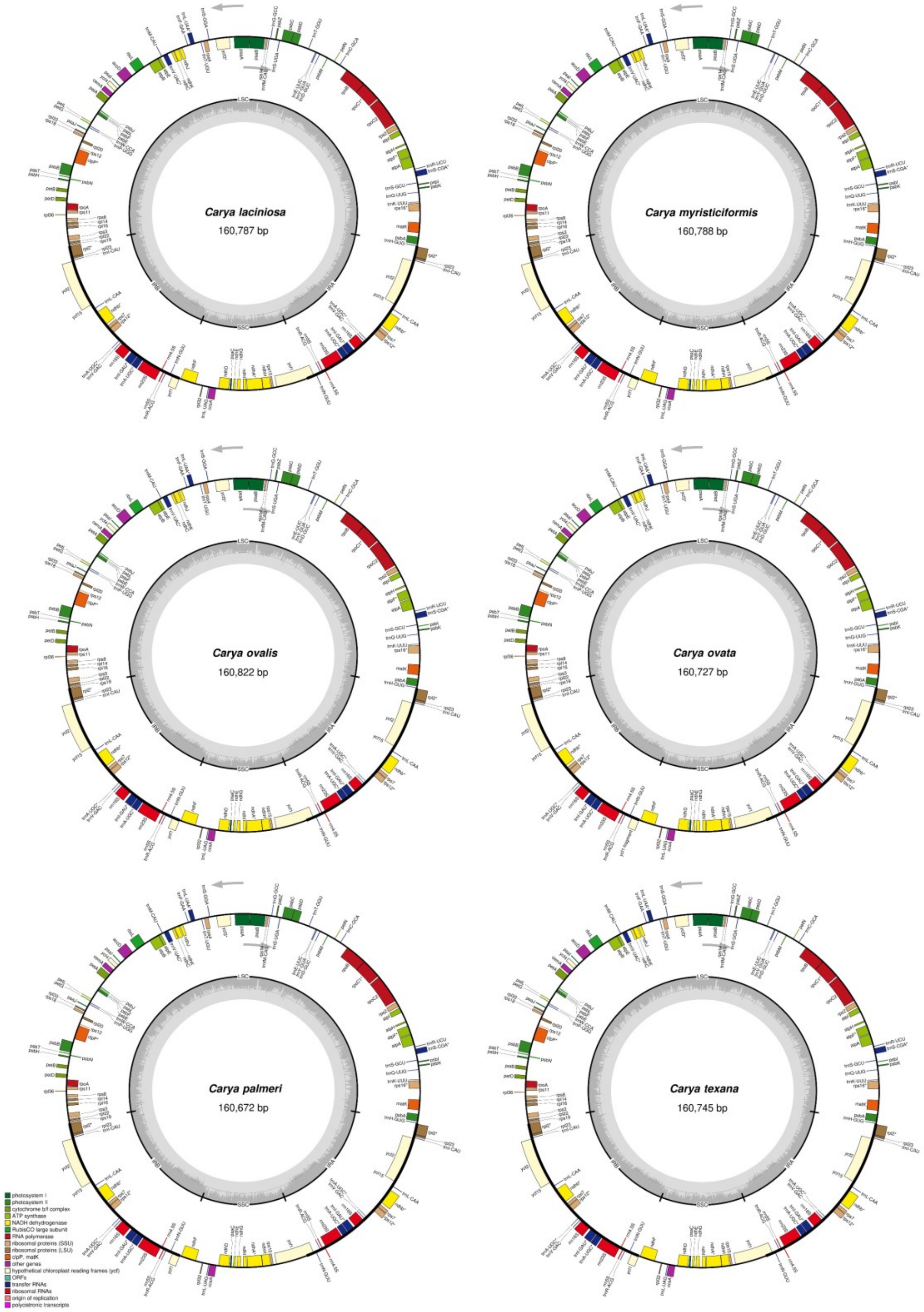
Carya laciniosa
160,787 bp
Carya myristiciformis
160,788 bp
Carya ovalis
160,822 bp
Carya ovata
160,727 bp
Carya palmeri
160,672 bp
Carya texana
160,745 bp
LSC
IRA
IRB
SSC
photosystem I
photosystem II
cytochrome b/f complex
ATP synthase
NADH dehydrogenase
RubisCO large subunit
RNA polymerase
ribosomal proteins (SSU)
ribosomal proteins (LSU)
clpP, matK
other genes
hypothetical chloroplast reading frames (ycf)
ORFs
transfer RNAs
ribosomal RNAs
origin of replication
polycistronic transcripts

Continued.

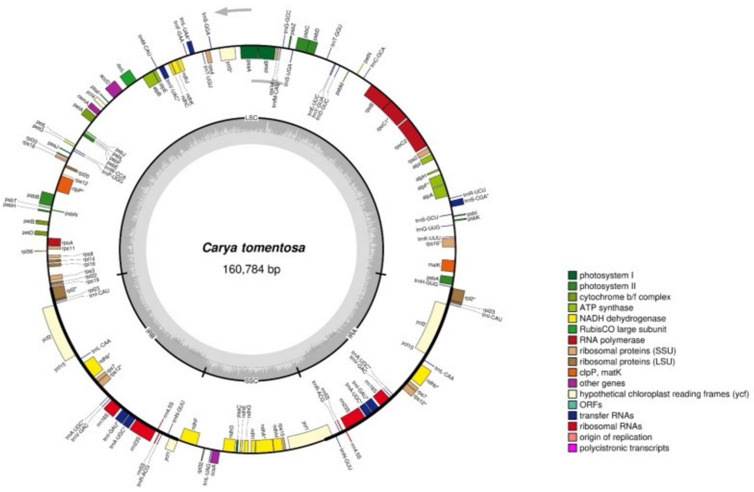

Carya tomentosa
160,784 bp
LSC
SSC
IRA
IRB
photosystem I
photosystem II
cytochrome b/f complex
ATP synthase
NADH dehydrogenase
RubisCO large subunit
RNA polymerase
ribosomal proteins (SSU)
ribosomal proteins (LSU)
clpP, matK
other genes
hypothetical chloroplast reading frames (ycf)
ORFs
transfer RNAs
ribosomal RNAs
origin of replication
polycistronic transcripts

Supplementary Figure 3. Validation of IR-SC boundaries by PCR amplification. A, validation of IRb-SSC boundary of *C. kweichowensis* using primers for amplification of the fragment *ycf1~ndhF*. B, Validation of IRb-SSC boundary of *C.* tonkinensis by amplification of the fragment *rrn23S~ndhF*. C, validation for all IR-SC boundaries for *C. cathayensis* (channels 1-4), *C. dabieshanensis* (channels 5-8), *C. hunanensis* (channels 9-12), *C. kweichowensis* (channels 13-16), *C. sinensis* (channels 17-20), *C. illinoinensis* 'sioux' (channels 21-24) and *C. tonkinensis* (channels 25-28). IRa-LSC boundary = channels 1, 5, 9, 13, 17, 21 and 25; LSC-IRb boundary = channels 2, 6, 10, 14, 18, 22 and 26; IRb-SSC boundary = channels 3, 7, 11, 15, 19, 23 and 27; SSC-IRa boundary = channels 4, 8, 12, 16, 20, 24 and 28. M, marker, the ladder lengths are 5000, 3000, 2000, 1500, 1000, 750, 500, 250, and 100 from up to down. The asterisks show the correct amplification and all the primers listed in Table S3.

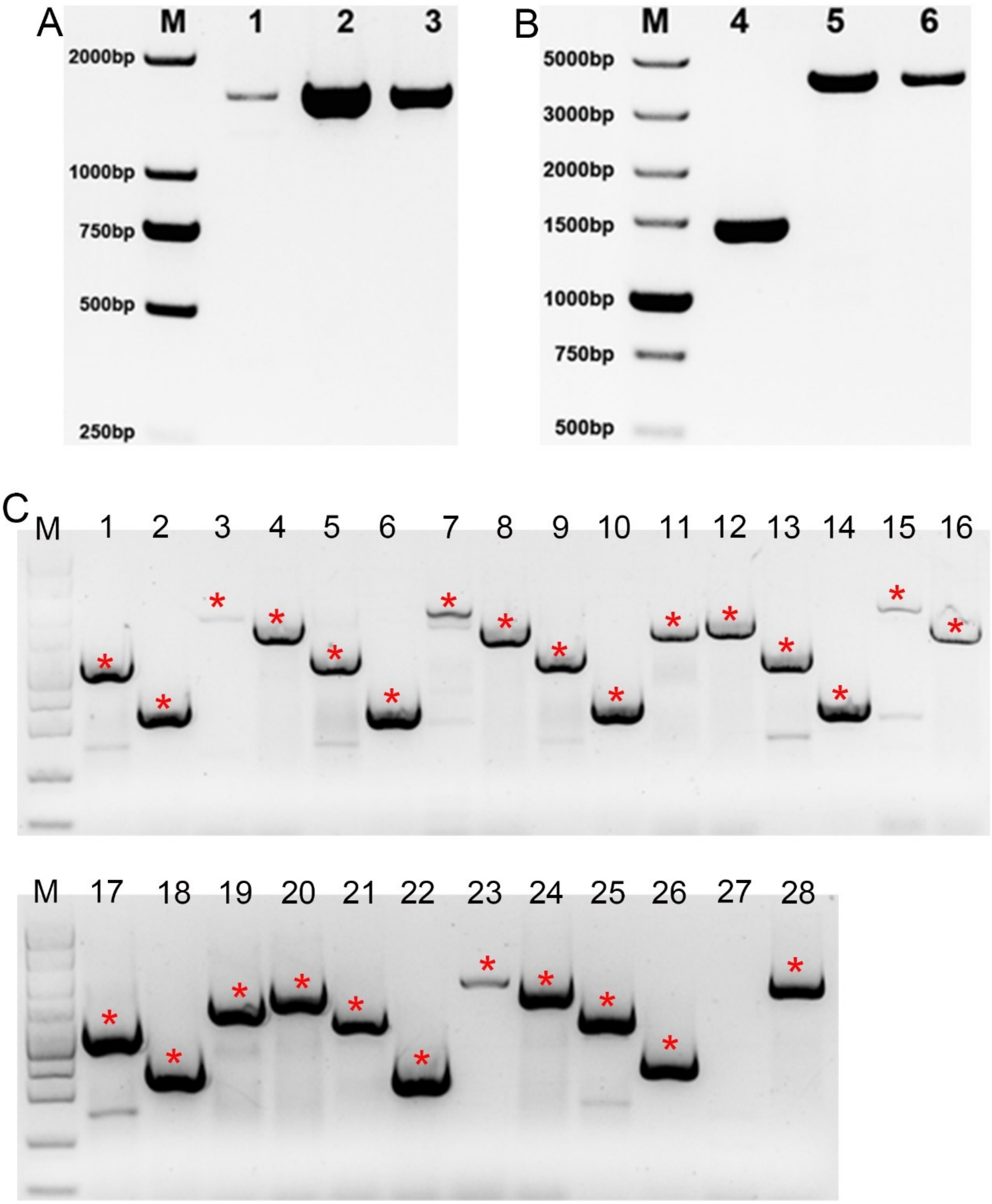

Supplementary Figure 4. Statistics of SSR types. A, SSR types and their proportions in *Carya* plastomes. B, The detailed statistics of SSR types in *Carya* plastomes.

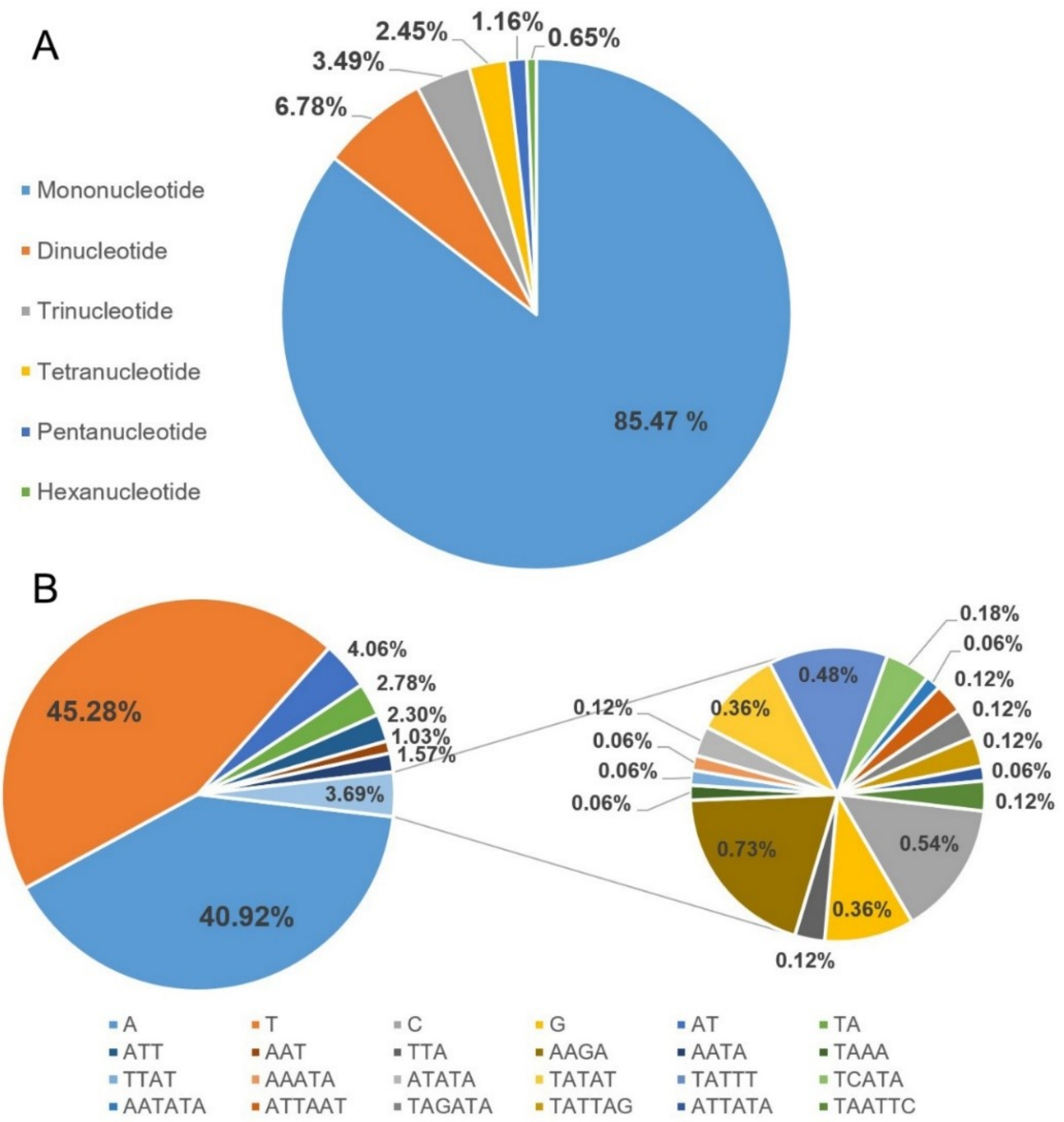

Supplementary Figure 5. Proportion of amino acids in *Carya* plastomes.

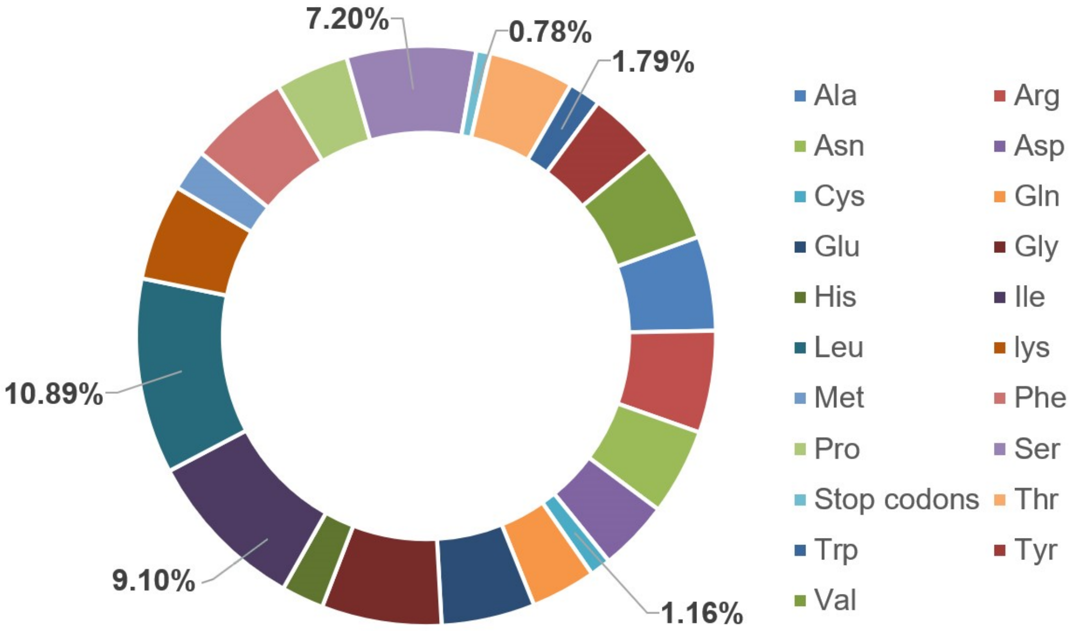

Supplementary Figure 6. Codon number (A) and usage statistics (B) of *Carya* plastomes.

(A)

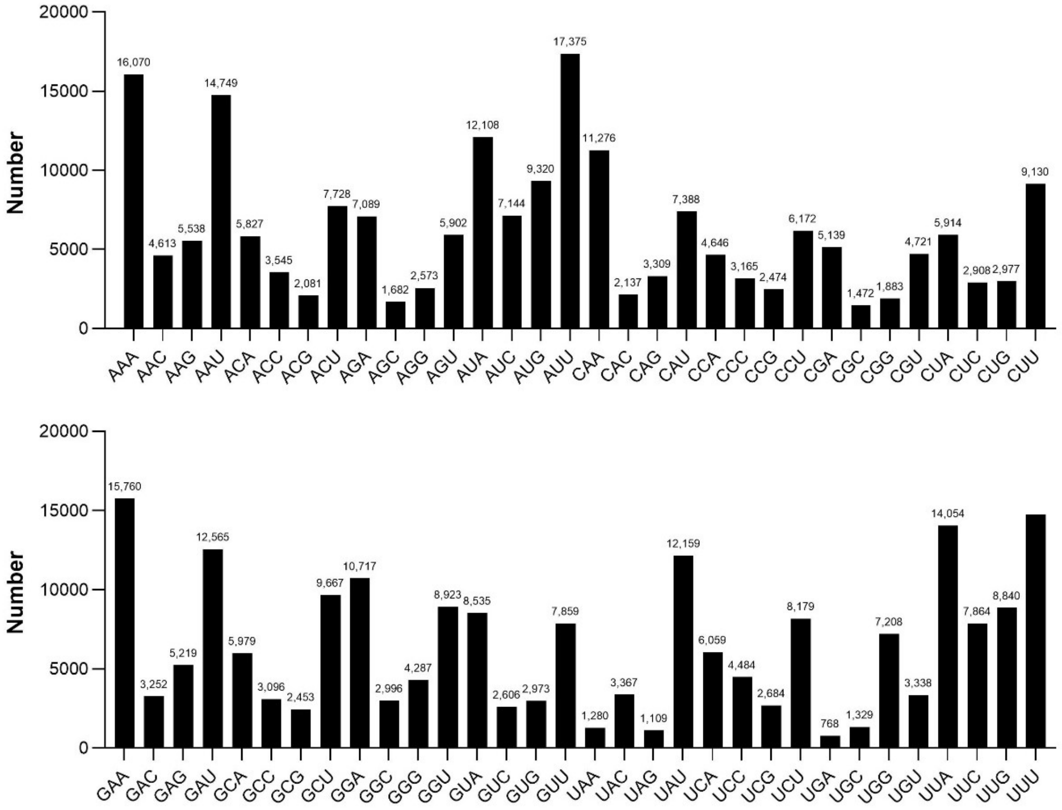

(B)

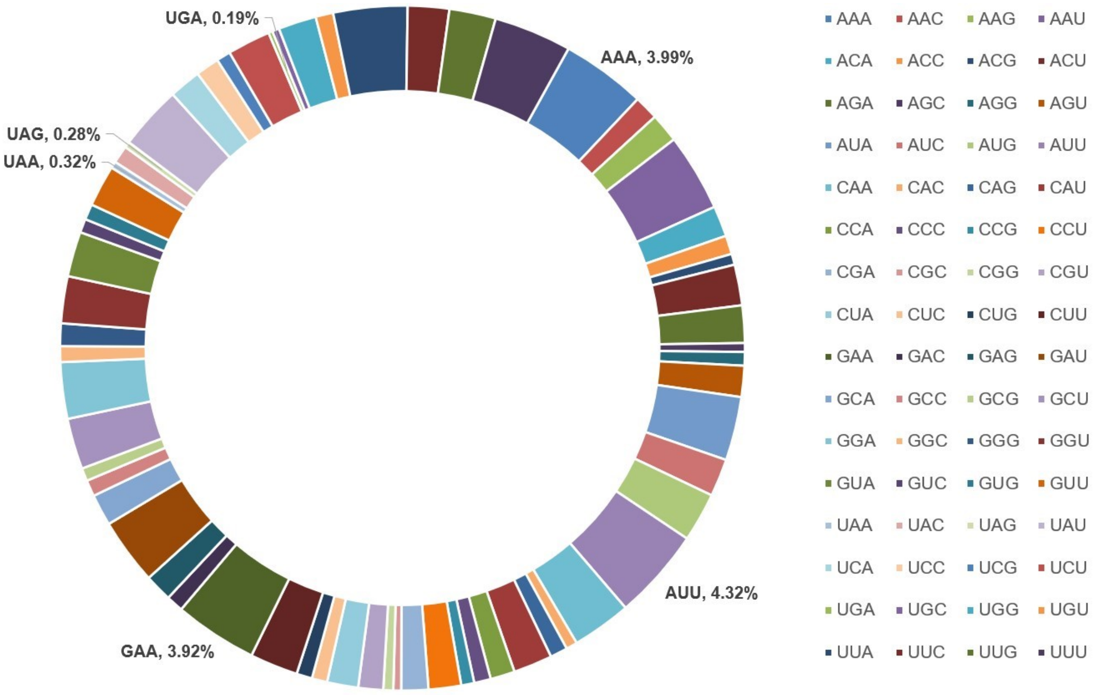

Supplementary Figure 7. Codon RSCU value of *Carya* plastomes.

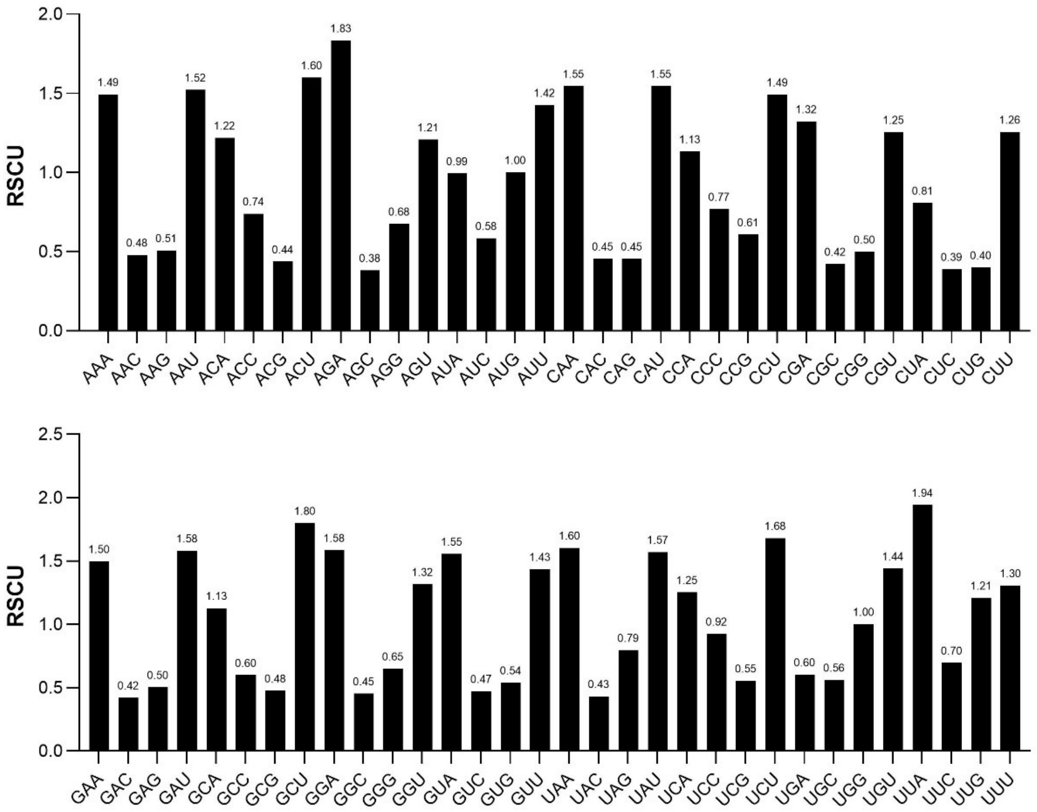

Supplementary Figure 8. RNA editing sites in the protein-coding genes of *Carya* plastomes. The genes with relatively large number, including *ndhB*, *ndhD*, *rpoB*, and *rpoC2*, were highlighted by different blue grid fill.

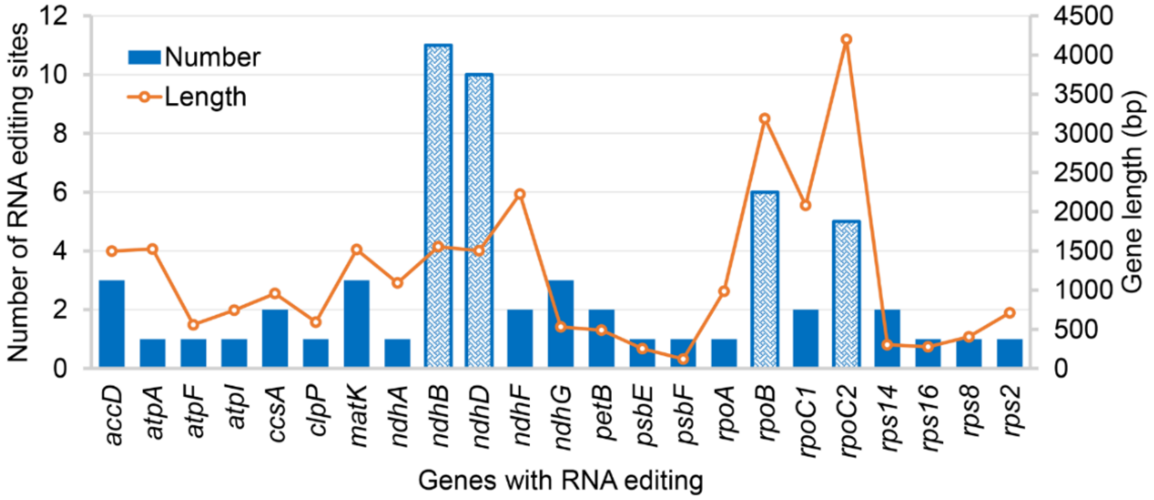

Supplementary Figure 9. Comparison of *Ka*/*Ks* (ω) values of protein-coding genes in *Carya* plastomes. Only the ω values not equal to zero are shown.

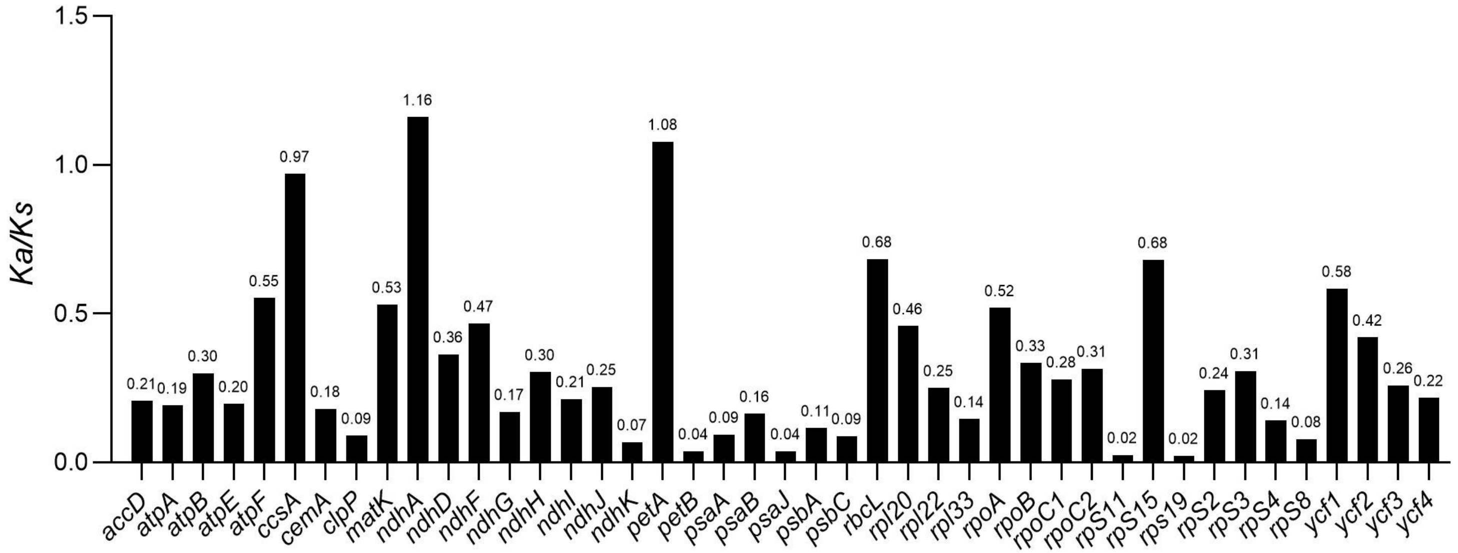

Supplementary Figure 10 . Phylogenetic trees of *Carya* species and representative species in other genera of Juglandaceae inferred from the datasets of LSC regions using Bayesian Inference (BI) and Maximum likelihood methods. The posterior probability (PP) and bootstrap (BS) values that supported each node are shown above the branches. The colors in green, light purple and pink indicate the outgroups, EA and NA *Carya* species, respectively.

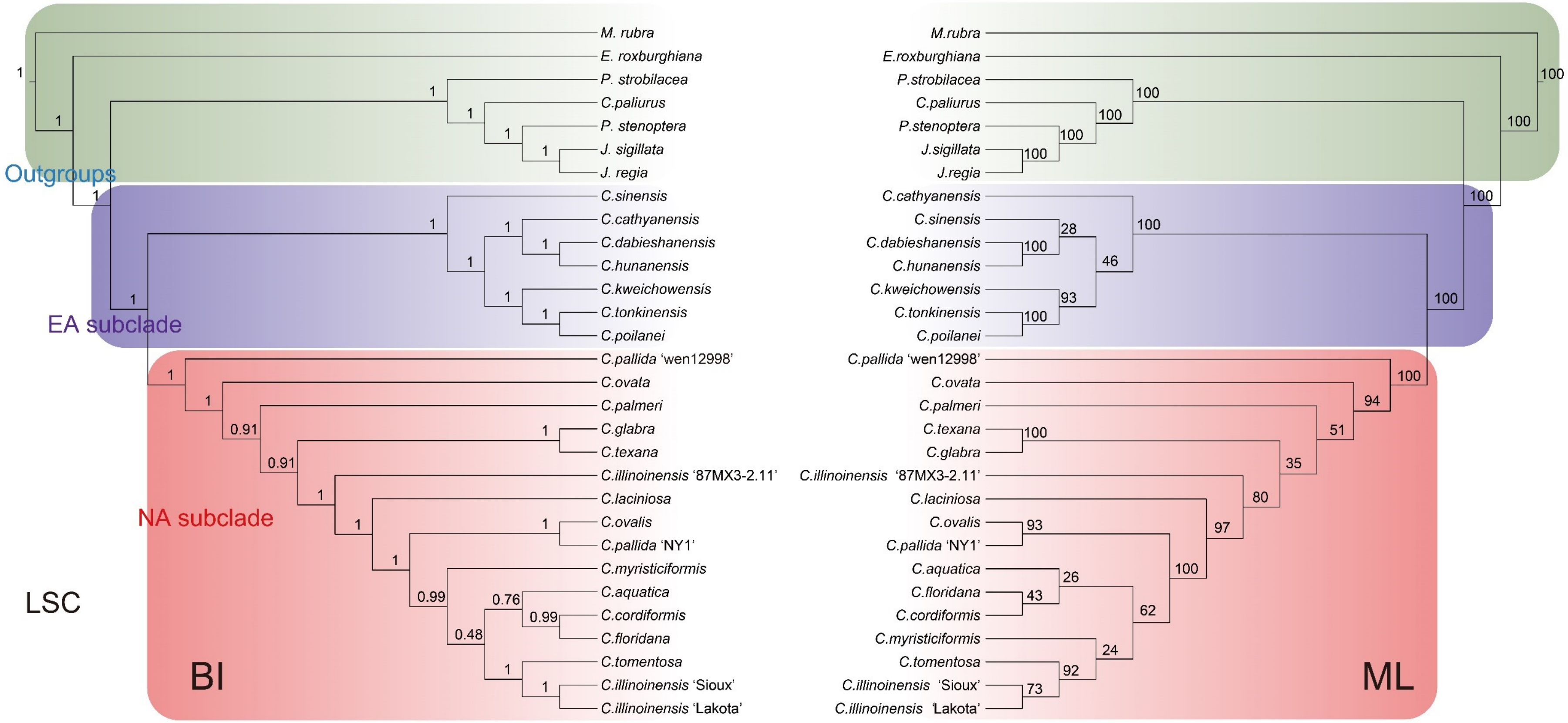

Supplementary Figure 11. Phylogenetic trees of *Carya* species and representative species in other genera of Juglandaceae inferred from the datasets of LSC-SSC regions using Bayesian Inference (BI) and Maximum likelihood methods. The posterior probability (PP) and bootstrap (BS) values that supported each node are shown above the branches. The colors in green, light purple and pink indicate the outgroups, EA and NA *Carya* species, respectively.

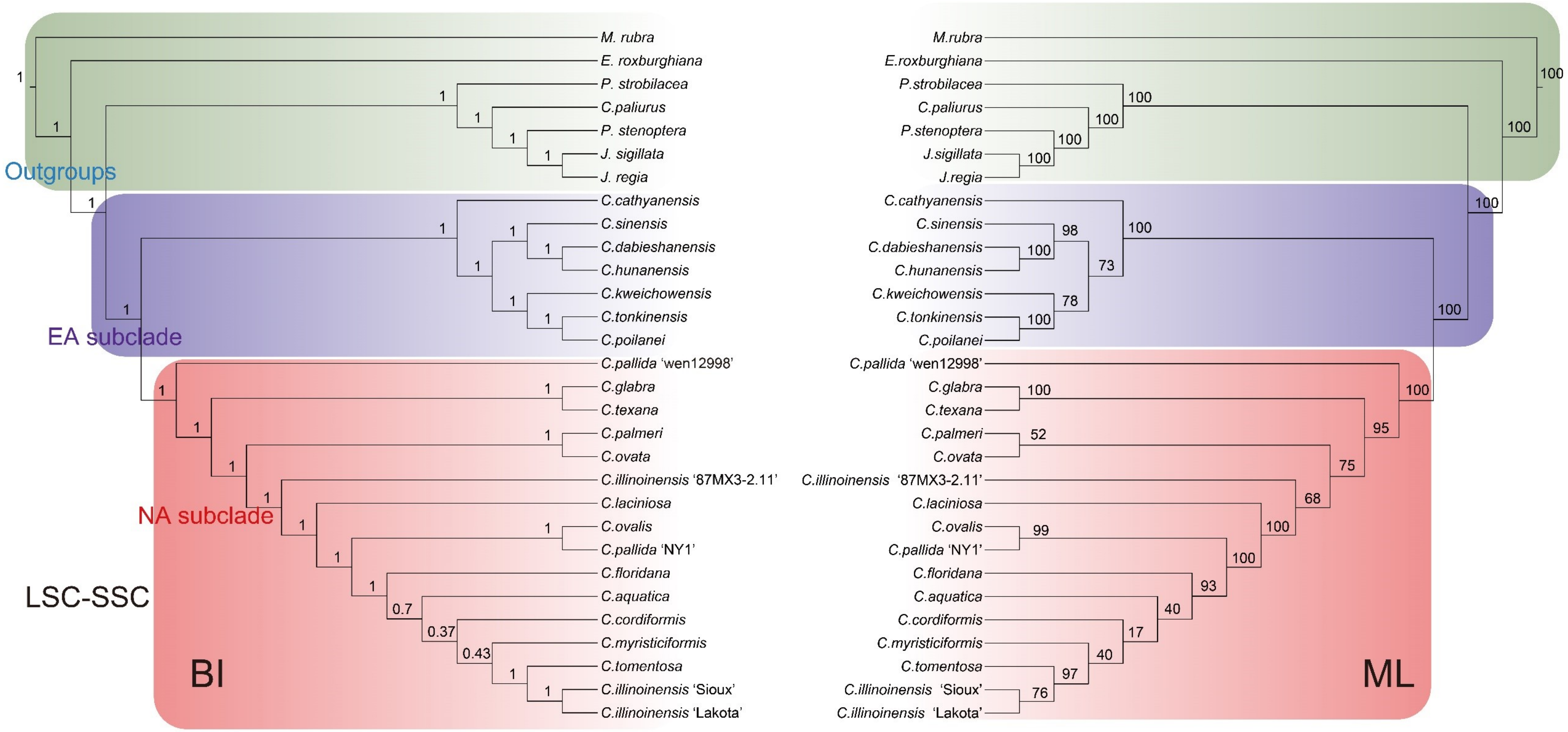

Supplementary Figure 12. Phylogenetic trees of *Carya* species and representative species in other genera of Juglandaceae inferred from the datasets of SSC regions using Bayesian Inference (BI) and Maximum likelihood methods. The posterior probability (PP) and bootstrap (BS) values that supported each node are shown above the branches. The colors in green, light purple and pink indicate the outgroups, EA and NA *Carya* species, respectively.

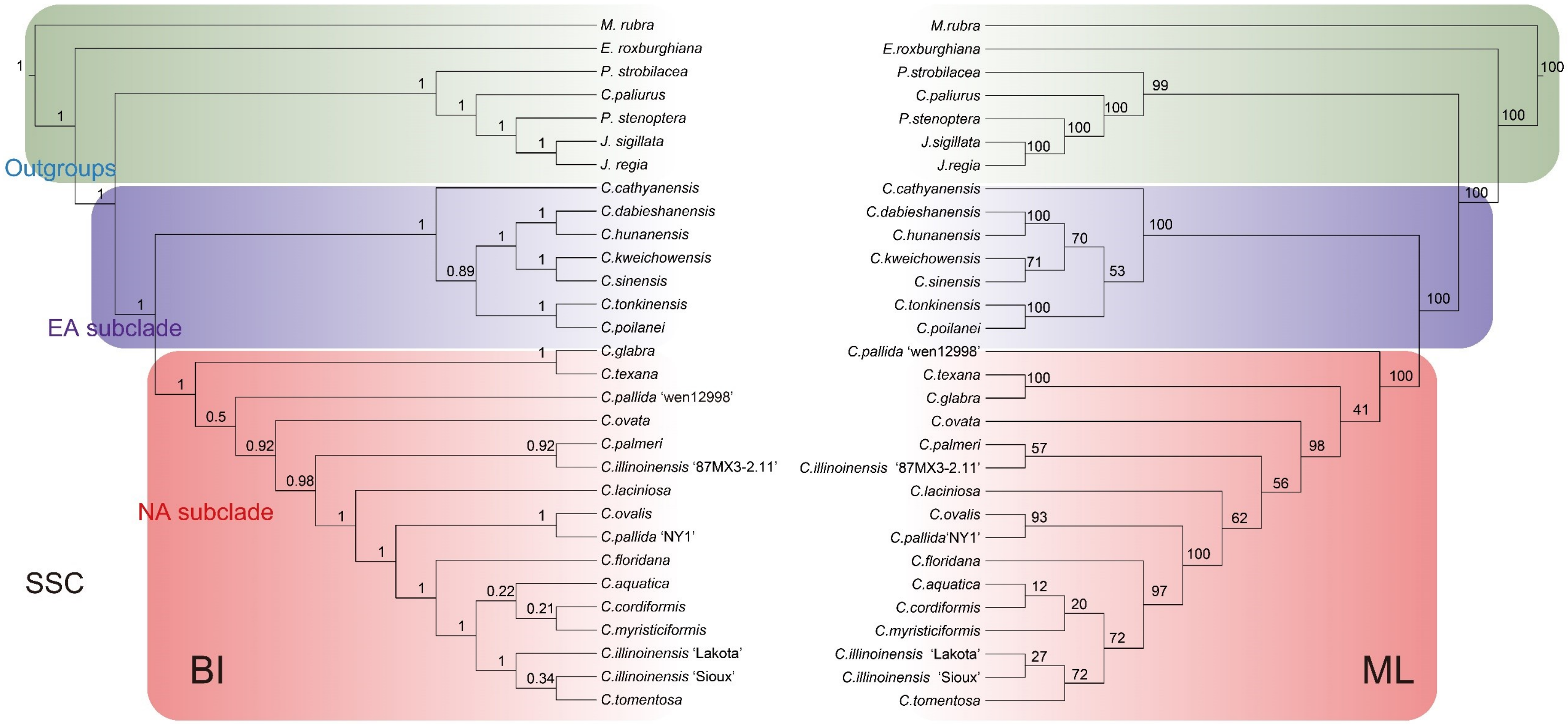

Supplementary Figure 13. Phylogenetic trees of *Carya* species and representative species in other genera of Juglandaceae inferred from the datasets of CDS regions using Bayesian Inference (BI) and Maximum likelihood methods. The posterior probability (PP) and bootstrap (BS) values that supported each node are shown above the branches. The colors in green, light purple and pink indicate the outgroups, EA and NA *Carya* species, respectively.

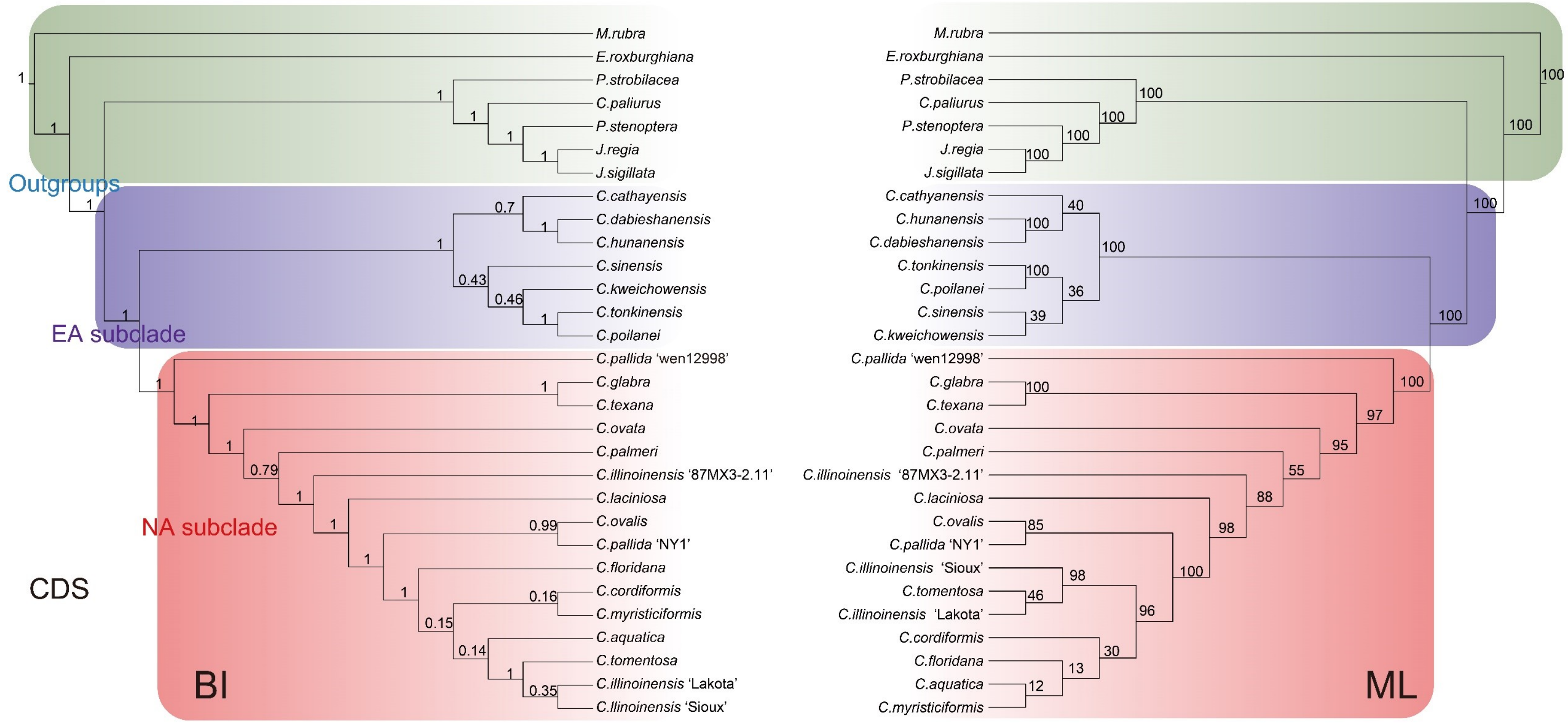

Supplementary Figure 14. Phylogenetic trees of *Carya* species and representative species in other genera of Juglandaceae inferred from the datasets of LSC-IR-SSC regions Bayesian Inference (BI) and Maximum likelihood methods. The posterior probability (PP) and bootstrap (BS) values that supported each node are shown above the branches. The colors in green, light purple and pink indicate the outgroups, EA and NA *Carya* species, respectively.

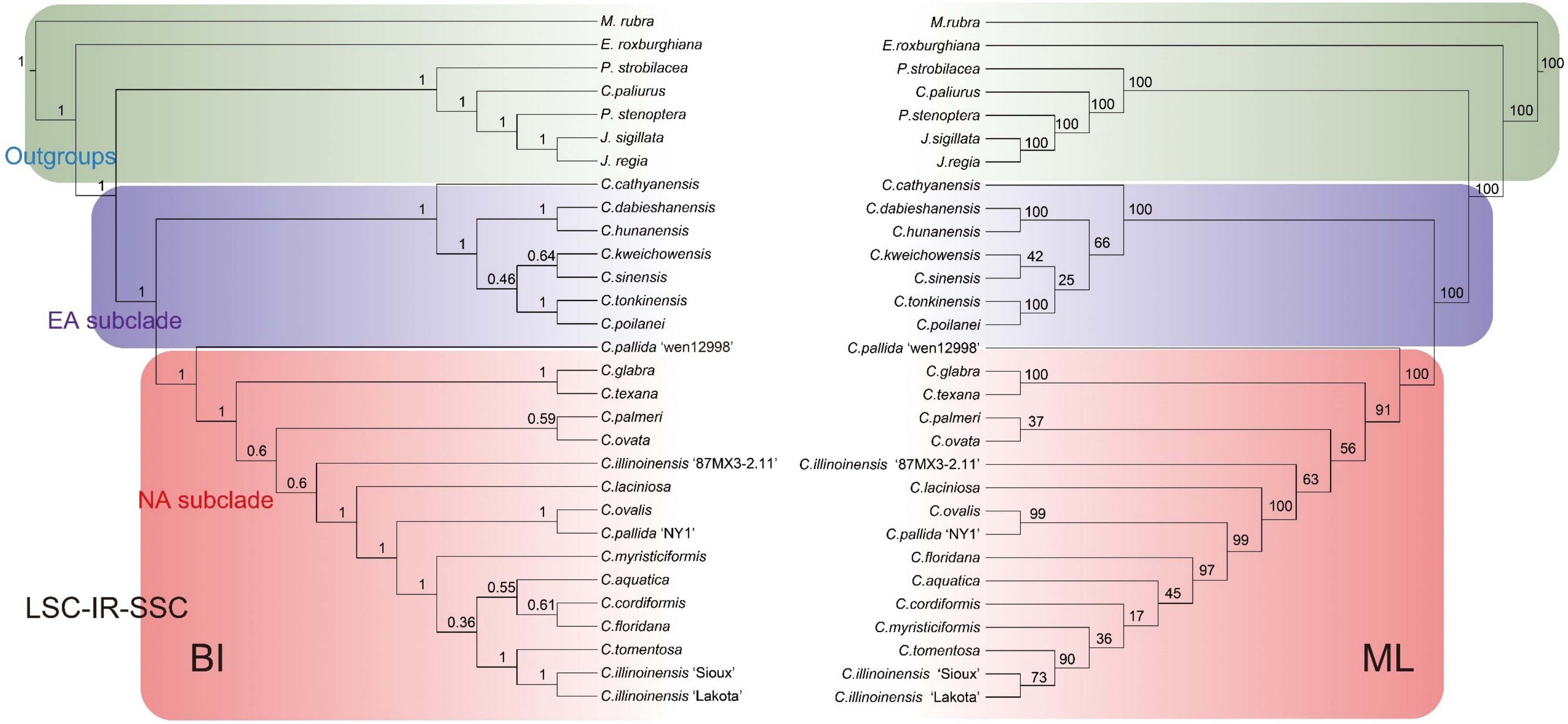

Supplementary Figure 15. Phylogenetic trees of *Carya* species and representative species in other genera of Juglandaceae inferred from the datasets of IR regions using Bayesian Inference (BI) and Maximum likelihood methods. The posterior probability (PP) and bootstrap (BS) values that supported each node are shown above the branches. The colors in green, light purple and pink indicate the outgroups, EA and NA *Carya* species, respectively.

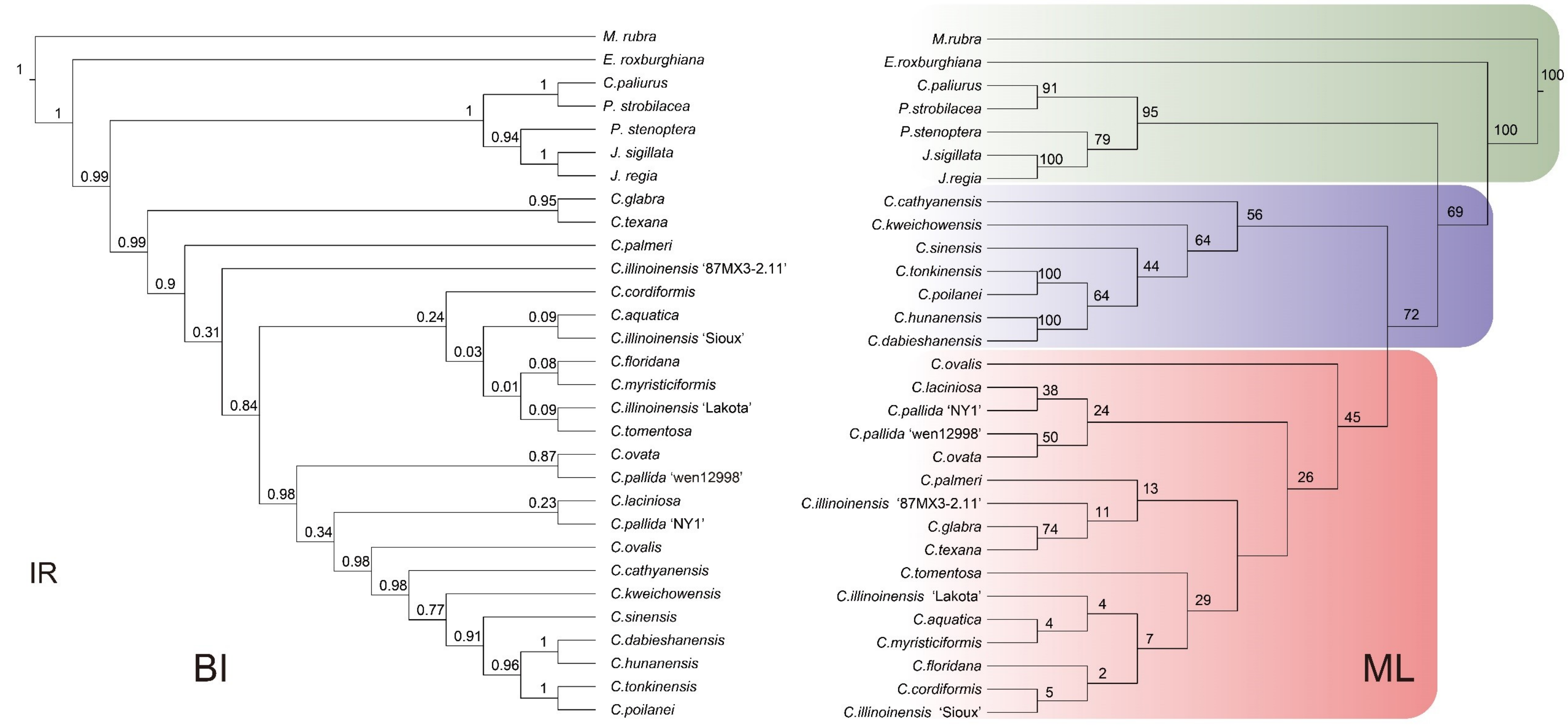

Supplementary Figure 16. The native geographical distributions of extant *Carya* species.

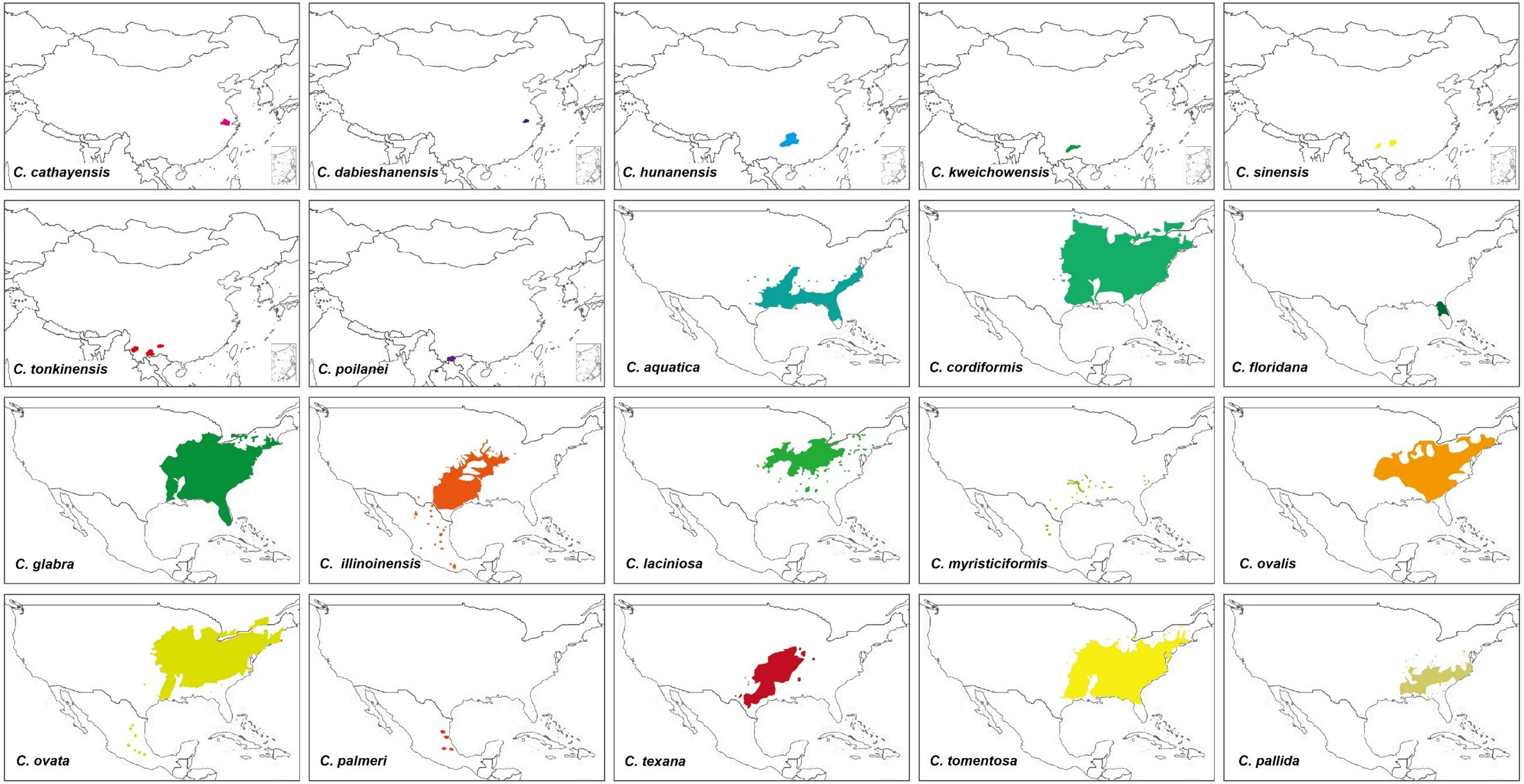

Supplementary Figure 17. Morphological features of *C. cathayensis* and *C. dabieshanensis*. (A), Nuts of *C. cathayensis.* (B), Nuts of *C. dabieshanensis.* (C) - (D), Statistics of fruit and nut indexes of *C. cathayensis* and *C. dabieshanensis.* The nut size was calculated from 10 trees with n = 20 fruits or nuts per three.

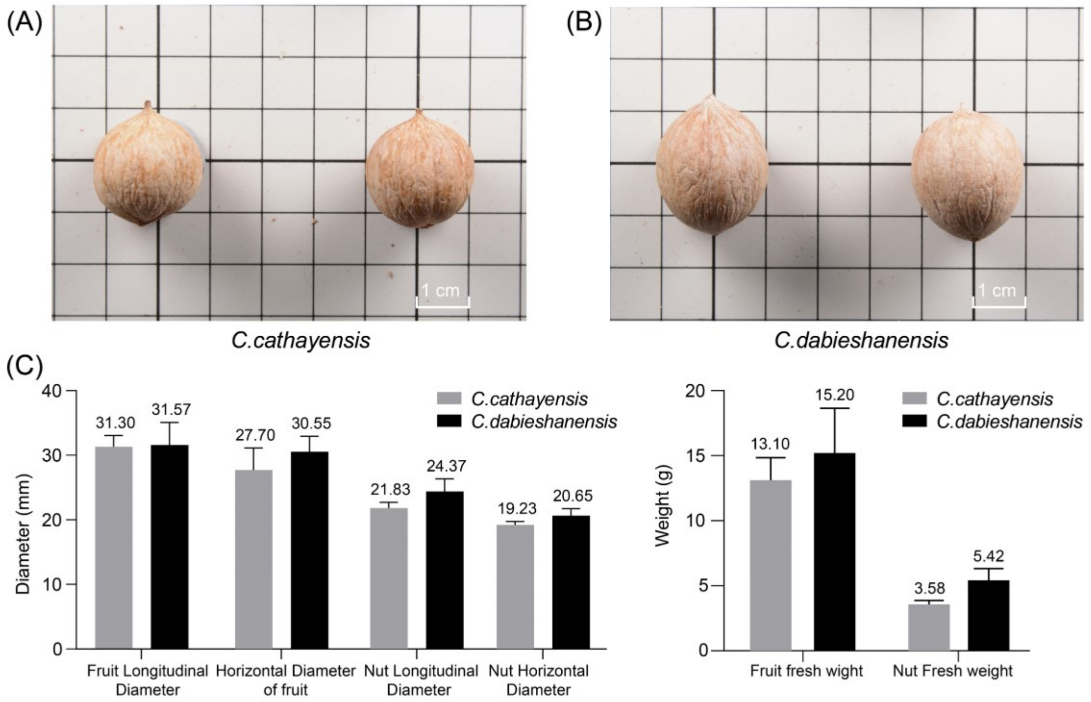

(D)

Item	*C. dabieshanensis*	*C. cathayensis*
Fruit fresh wight/g	15.2±3.437	13.10±1.74
Fruit Longitudinal Diameter (mm)	31.57±3.512	31.30±3.41
Horizontal Diameter of fruit (mm)	30.55±2.388	27.70±3.05
Nut Fresh weight (g)	5.42±0.904	3.58±0.30
Nut Longitudinal Diameter (mm)	24.37±1.979	21.83±0.88
Nut Horizontal Diameter (mm)	20.65±1.061	19.23±0.53

Supplementary Tables

Supplementary Table 1. Information on sample collection and data sources for plastome assemblies of *Carya*.

species	Sampling site	longitude	latitude	altitude (m)	NCBI ID number	String length
C. cathyanensis	Orchard in Zhejiang A&F University in Linan District, Zhejiang Province	119°43'42"	30°15'16"	38.0	NA	29
C. dabieshanensis	Yanzihe Village Jinzhai District, Anhui Province	115°49′24″	31°16′22″	712.5	NA	49
C. hunanensis	Orchard in Zhejiang A&F University in Linan District, Zhejiang Province	119°43'42"	30°15'16"	38.0	NA	19
C. kweichowensis	Qiannan Buyi and Miao Autonomous Prefecture, Guizhou Province	107°53'47"	25°11'53"	820.0	NA	39
C. sinensis	Qiannan Buyi and Miao Autonomous Prefecture, Guizhou Province	106°44′4″	25°37′40″	1000.6	NA	19
C. tonkinensis	Conservatory in Zhejiang A&F University in Linan District, Zhejiang Province	119°43'15"	30°15'55"	38.0	NA	29
C. illinoinensis 'Sioux'	Orchard in Zhejiang A&F University in Linan District, Zhejiang Province	119°43'42"	30°15'16"	38.0	NA	40
C. poilanei	Jianshui County, Yunnan Province	102°51'36"	23°23'42"	1895.0	SRX13427070	39
C. aquatica	—	—	—	—	SRR6804841	29
C. cordiformis	—	—	—	—	SRR6804840	39
C. floridana	—	—	—	—	SRR6804844	49
C. glabra	—	—	—	—	SRR6804848	29
C. laciniosa	—	—	—	—	SRR6804855	29
C. myristiciformis	—	—	—	—	SRR6804845	39
C. ovalis	—	—	—	—	SRR6804843	19
C. ovata	—	—	—	—	SRR6804846	39
C. palmeri	—	—	—	—	SRR6804847	39
C. texana	—	—	—	—	SRR6804850	49
C. tomentosa	—	—	—	—	SRR6804842	39

Supplementary Table 2. Primers for validation of IR-SC boundaries and *Carya* plastome assemblies.

IR-SC boundary	Forward primer sequence (5'-3')	Forward primer sequence (5'-3')
ycf1~ndhF	AATCGAGAAGAAAGTATCAGCAA	TGGATTGGTATGAATTTGTGAC
rrn23S~ndhF	AAAAGTTACTCTAGGGATAACAGG	TGGATTGGTATGAATTTGTGAC
IRa-LSC	CACGGCCATACATAACATAGAAATC	GTTCTTTACATTTCTTCCTAGCGG
LSC-IRb	GCTCGGGACCAAGTTATTATTATTTC	CACGGCCATACATAACATAGAAATC
Irb-SSC	CTCCGAGCTCGGATTATAGAAGAAG	TGGATTGGTATGAATTTGTGAC
SSC-IRa	GTAATTGGTAGTAAGAAGGATGCC	CTCCGAGCTCGGATTATAGAAGAAG

Supplementary Table 3. Species information used for phylogeny analysis.

Family	Species	GenBank accession
Juglandaceae	*Carya cathayensis*	MW410227
	Carya aquatica	MW255965
	Carya cordiformis	MW368387
	Carya dabieshanensis	MW410228
	Carya floridana	MW410229
	Carya glabra	MW410230
	Carya hunanensis	MW298527
	Carya illinoinensis ‘sioux’	MW410238
	Carya kweichowensis	MW410237
	Carya laciniosa	MW410231
	Carya myristiciformis	MW410232
	Carya ovalis	MW440674
	Carya ovata	MW410233
	Carya palmeri	MW410234
	Carya sinensis	MW421595
	Carya pallinda ‘NY1’	MT554070
	Carya pallinda ‘Wen12998’	MT554071
	Carya texana	MW410235
	Carya tomentosa	MW410236
	Carya tonkinensis	MW368388
	Carya poilanei	ON568300
	Juglans regia	NC_028617.1
	Juglans sigillata	NC_031373.1
	Pterocarya stenoptera	MN262640.1
	Platycarya strobilacea	NC_035413.1
	Cyclocarya paliurus	NC_034315.1
	Engelhardia roxburghiana	NC_046434.1
	Carya sinensis	KX703001.1
	Carya illinoinensis ‘87MX3-2.11’	MH909600.1
	Carya illinoinensis ‘Lakota’	MH909599.1
Myricaceae	*Myrica rubra*	NC_035006.1

Supplementary Table 4. Summary of intron-containing genes in *Carya* plastomes.

Gene	Region	Exon I length (bp)	Intron I length (bp)	Exon II length (bp)	Intron II length (bp)	Exon III length (bp)
atpF	LSC	411	(759~764)[a], (747~755)[b]	147,144[c]	-	-
clpP	LSC	228	(611~615)[a], (609~620)[b]	294	(848~851)[a], (847~855)[b]	69
ndhA	SSC	537	(1186~1121)[a], (1167~1229)[b]	555	-	-
ndhB	IR	792	671	762	-	-
rpl2	IR	434[a], 471[b]	(663~692)[a], (656~662)[b]	391[a], 390[b]	-	-
rpoC1	LSC	1626	(819~831)[a], (811~814)[b]	456	-	-
rps12	LSC-IR	114	537	231	-	30
rps16	LSC	234, 225[d]	(888~897)[a], (896~904)[b]		-	-
ycf3	LSC	155[a], 161[b]	(792~796)[a], (790~791)[b]	226[a], 223[b]	720~721	126
trnA-UGC	IR	38	(805~808)[a], 807[b]	35	-	-
trnI-GAU	IR	37	955	35	-	-
trnL-UAA	LSC	35	526	50	-	-
trnS-CGA	LSC	23	(724~726)[a], (685~725)[b]	38	-	-
trnV-UAC	LSC	37	617	36	-	-

Note: a, Exist in EA species; b, Exist in NA species; c, Exon II length of *atpF* is 144 bp in *C. dabieshanensis*; d, Exon I length of *rps16* is 225 bp in *C. tonkinensis*.

Supplementary Table 5. The numbers and repeat sequences of each SSR type in *Carya* plastomes.

Species	Number							Repeat sequence type
	P1	P2	P3	P4	P5	P6	Total	
C. cathayensis	85	3	2	2	1	0	93	A/T/C/G/AT/TA/ATT/AATA/AAATA
C. dabieshanensis	89	6	4	2	2	2	105	A/T/G/AT/TA/AAT/ATT/TTA/AATA/TATAT/TCATATAATTC/TATTAG
C. hunanensis	87	6	4	2	2	2	103	A/T/AT/TA/AAT/ATT/TTA/AATA/TATAT/TCATA/TAATTC/TATTAG
C. kweichowensis	82	5	3	2	0	0	92	A/T/C/AT/TA/AAT/ATT/AATA
C. sinensis	89	6	2	2	2	0	101	A/T/C/G/AT/TA/ATT/AATA/ATATA/TCATA
C. tonkinensis	91	8	3	2	2	0	106	A/T/C/G/AT/TA/AAT/ATT/AATA/TATAT
C. poilanei	91	8	3	2	2	0	106	A/T/C/G/AT/TA/AAT/ATT/AATA/TATAT
C. aquatica	64	6	3	2	1	0	76	A/T/AT/TA/AAT/ATT/AAGA/AATA/TATTT
C. cordiformis	66	6	3	2	1	1	79	A/T/AT/TA/AAT/ATT/AAGA/AATA/TATTT/ATTAAT
C. floridana	67	6	3	2	1	0	79	A/T/AT/TA/AAT/ATT/AAGA/AATA/TATTT
C. glabra	66	6	3	2	0	0	77	A/T/AT/TA/AAT/ATT/AAGA/AATA
C. illinoinensis 'Sioux'	66	5	3	2	1	1	78	A/T/AT/TA/AAT/ATT/AAGA/AATA/TATTT/TAGATA
C. laciniosa	65	7	3	3	1	0	79	A/T/G/AT/TA/AAT/ATT/AAGA/AATA/TTAT/TATTT
C. myristiciformis	66	6	3	2	1	0	78	A/T/AT/TA/AAT/ATT/AAGA/AATA/TATTT
C. ovalis	70	6	3	2	1	0	82	A/T/AT/TA/AAT/ATT/AAGA/AATA/TATTT
C. ovata	68	5	3	3	0	2	81	A/T/AT/TA/AAT/ATT/AAGA/AATA/ATTAAT/ATTATA
C. palmeri	69	6	3	2	1	1	82	A/T/C/AT/TA/AAT/ATT/AAGA/TAAAATATA/AATATA
C. texana	65	6	3	2	0	0	76	A/T/AT/TA/AAT/ATT/AAGA/AATA
C. tomentosa	66	6	3	2	1	1	79	A/T/AT/TA/AAT/ATT/AAGA/AATA/TATTT/TAGATA

Supplementary Table 6. Codon usage in plastomes of assemblies and *J. regia*.

Species	Codon number	ENC	Codon GC content (%)			
			Total	1st position	2nd position	3rd position
C. cathayensis	21,190	48.88	37.33	45.76	37.62	28.60
C. dabieshanensis	21,196	48.96	37.34	45.78	37.60	28.64
C. hunanensis	21,196	48.95	37.34	45.77	37.61	28.64
C. kweichowensis	21,204	48.93	37.35	45.80	37.63	28.62
C. sinensis	21,207	48.96	37.35	45.75	37.62	28.67
C. tonkinensis	21,206	56.51	37.32	39.59	31.12	41.26
C. poilanei	21,206	56.52	37.32	39.59	31.18	41.20
C. aquatica	21,210	48.98	37.37	45.82	37.59	28.70
C. cordiformis	21,210	48.97	37.37	45.82	37.60	28.70
C. floridana	21,210	48.98	37.38	45.83	37.60	28.70
C. glabra	21,210	48.97	37.38	45.82	37.61	28.70
C. illinoinensis 'Sioux'	21,210	48.98	37.38	45.82	37.60	28.70
C. laciniosa	21,210	48.93	37.35	45.80	37.63	28.62
C. myristiciformis	21,210	48.98	37.37	45.82	37.60	28.70
C. ovalis	21,208	48.98	37.38	45.83	37.59	28.71
C. ovata	21,210	49.00	37.39	45.81	37.61	28.74
C. palmeri	21,210	48.99	37.38	45.82	37.60	28.72
C. texana	21,210	48.96	37.35	45.75	37.62	28.67
C. tomentosa	21,210	48.98	37.38	45.82	37.60	28.71
J. regia	21,265	48.78	37.20	45.77	37.49	28.33

Note: ENC, Effective Number of Codon.

Supplementary Table 7. Predicted RNA-editing sites in the protein-coding genes of *Carya* plastomes.

No.	Gene	Nucleotide position	Amino acid position	Codon change	Amino acid change	value	Note
1	*accD*	154	52	CGG→UGG	R→W	1	19 species
2	*accD*	775	259	CAU→UAU	H→Y	0.8	19 species
3	*accD*	815	272	UCG→UUG	S→L	0.8	19 species
4	*atpA*	914	305	UCA→UUA	S→L	1	19 species
5	*atpF*	92	31	CCA→CUA	P→L	0.86	19 species
6	*atpI*	211	71	CUC→UUC	L→F	1	only in *C. poilanei*
7	*atpI*	629	210	UCA→UUA	S→L	1	19 species
8	*ccsA*	494	165	UCU→UUU	S→F	0.86	Not found in *C. tonkenensis* and *C. poilanei*
9	*ccsA*	500	167	UCU→UUU	S→F	0.86	Only in *C. tonkenensis* and *C. poilanei*
10	*clpP*	559	187	CAU→UAU	H→Y	1	19 species
11	*matK*	640	214	CAU→UAU	H→Y	1	19 species
12	*matK*	1177	393	CGG→UGG	R→W	1	Not found in *C. cathayensis*
13	*matK*	1183	395	CGG→UGG	R→W	1	Only in *C. sinensis*
14	*ndhA*	1028	343	UCU→UUU	S→F	1	Only in *C. tonkenensis* and *C. poilanei*
15	*ndhB*	149	50	UCA→UUA	S→L	1	19 species
16	*ndhB*	467	156	CCA→CUA	P→L	1	19 species
17	*ndhB*	586	196	CAU→UAU	H→Y	1	19 species
18	*ndhB*	611	204	UCA→UUA	S→L	0.8	19 species
19	*ndhB*	737	246	CCA→CUA	P→L	1	19 species
20	*ndhB*	746	249	UCU→UUU	S→F	1	19 species
21	*ndhB*	845	282	UCA→UUA	S→L	1	19 species
22	*ndhB*	851	284	UCA→UUA	S→L	1	19 species

23	*ndhB*	1127	376	UCA→UUA	S→L	1	19 species
24	*ndhB*	1270	424	CAU→UAU	H→Y	1	19 species
25	*ndhB*	1502	501	CCA→CUA	P→L	1	19 species
26	*ndhD*	2	1	ACG→AUG	U→M	1	19 species
27	*ndhD*	305	102	UCA→UUA	S→L	1	19 species
28	*ndhD*	313	105	CGG→UGG	R→W	0.8	19 species
29	*ndhD*	383	128	UCA→UUA	S→L	1	19 species
30	*ndhD*	599	200	UCA→UUA	S→L	1	19 species
31	*ndhD*	674	225	UCA→UUA	S→L	1	19 species
32	*ndhD*	878	293	UCA→UUA	S→L	1	19 species
33	*ndhD*	887	296	CCC→CUC	P→L	1	19 species
34	*ndhD*	1298	433	UCA→UUA	S→L	0.8	19 species
35	*ndhD*	1405	469	CUU→UUU	L→F	0.8	19 species
36	*ndhF*	586	196	CUU→UUU	L→F	0.8	19 species
37	*ndhF*	1780	594	CCA→UCA	P→S	1	Only in *C. floridana*
38	*ndhG*	166	56	CAU→UAU	H→Y	0.8	19 species
39	*ndhG*	314	105	ACA→AUA	U→I	0.8	19 species
40	*ndhG*	494	165	GCU→GUU	A→V	0.8	19 species
41	*petB*	373	125	CCC→UCC	P→S	1	Only in *C. dabieshanensis* and *C. hunanensis*
42	*petB*	452	151	CCA→CUA	P→L	1	19 species
43	*psbE*	214	72	CCU→UCU	P→S	1	19 species
44	*psbF*	77	26	UCU→UUU	S→F	1	19 species
45	*rpoA*	830	277	UCA→UUA	S→L	1	19 species
46	*rpoB*	314	105	UCU→UUU	S→F	1	19 species
47	*rpoB*	527	176	UCA→UUA	S→L	1	19 species

48	*rpoB*	542	181	UCG→UUG	S→L	1	19 species
49	*rpoB*	586	196	CUU→UUU	L→F	1	Not found in *C. sinensis* and *C. kweichowensis*
50	*rpoB*	1976	659	UCU→UUU	S→F	1	19 species
51	*rpoB*	2402	801	UCA→UUA	S→L	0.86	19 species
52	*rpoC1*	62	21	UCA→UUA	S→L	1	19 species
53	*rpoC1*	278	93	UCU→UUU	S→F	1	19 species
54	*rpoC2*	1456	486	CGC→UGC	R→C	0.86	19 species
55	*rpoC2*	1597	533	CUU→UUU	L→F	1	19 species
56	*rpoC2*	2345	782	GCU→GUU	A→V	1	19 species
57	*rpoC2*	3146	1049	ACC→AUC	U→I	0.86	19 species
58	*rpoC2*	3764	1255	UCA→UUA	S→L	0.86	19 species
59	*rps14*	80	27	CCC→CUC	P→L	1	19 species
60	*rps14*	149	50	CCA→CUA	P→L	1	19 species
61	*rps16*	218	73	UCA→UUA	S→L	0.83	19 species
62	*rps8*	172	58	CUU→UUU	L→F	1	only in *C. laciniosa*
63	*rps2*	248	83	UCA→UUA	S→L	1	19 species

Supplementary Table 8. ***Ka*/*Ks* (*ω*) and nucleotide polymorphisms of protein-coding Gene in *Carya* plastomes.**

Gene	*Ka*/*Ks* (*ω*)	*π*	*θ*	Gene	*Ka*/*Ks* (*ω*)	*π*	*θ*
accD	0.206883	0.002260	0.002680	*psbH*	0.000000	0.000900	0.001290
atpA	0.191243	0.001340	0.002440	*psbI*	0.000000	0.001790	0.002580
atpB	0.299204	0.001400	0.000970	*psbJ*	0.000000	0.000000	0.000000
atpE	0.196416	0.001480	0.001420	*psbK*	0.000000	0.001070	0.001540
atpF	0.551268	0.002970	0.003110	*psbL*	0.000000	0.010100	0.007340
atpH	0.000000	0.000000	0.000000	*psbM*	0.000000	0.000000	0.000000
atpI	0.000000	0.001210	0.001540	*psbT*	0.000000	0.004550	0.002650
ccsA	0.969747	0.004290	0.005380	*psbZ*	0.000000	0.001610	0.003030
cemA	0.179279	0.001950	0.003730	*rbcL*	0.682693	0.001490	0.001800
clpP	0.089417	0.001350	0.001450	*rpl14*	0.000000	0.001970	0.004650
matK	0.530389	0.003300	0.005840	*rpl16*	0.000000	0.002220	0.002090
ndhA	1.160245	0.002630	0.002620	*rpl2*	0.000000	0.000000	0.000000
ndhB	0.000000	0.000070	0.000180	*rpl20*	0.457265	0.003800	0.004850
ndhC	0.000000	0.000000	0.000000	*rpl22*	0.249360	0.005990	0.008200
ndhD	0.362368	0.005420	0.004950	*rpl23*	0.000000	0.000000	0.000000
ndhE	0.000000	0.002600	0.002810	*rpl32*	0.000000	0.005410	0.005840
ndhF	0.466137	0.003260	0.003210	*rpl33*	0.144969	0.004350	0.005530
ndhG	0.169405	0.003480	0.003230	*rpl36*	0.000000	0.084950	0.223370
ndhH	0.302579	0.002720	0.002660	*rpoA*	0.518453	0.002330	0.002900
ndhI	0.213336	0.002480	0.002270	*rpoB*	0.332935	0.001760	0.002510
ndhJ	0.253986	0.002280	0.001800	*rpoC1*	0.278822	0.001260	0.002060
ndhK	0.067377	0.004210	0.003360	*rpoC2*	0.314354	0.002480	0.002870
pbf1	0.000000	0.004520	0.004340	*rpS11*	0.024313	0.002610	0.002060
petA	1.077751	0.002480	0.003260	*rpS12*	0.000000	0.000000	0.000000
petB	0.037075	0.001340	0.001760	*rpS14*	0.000000	0.003590	0.002830
petD	0.000000	0.001160	0.001090	*rpS15*	0.680318	0.002960	0.004190
petG	0.000000	0.000000	0.000000	*rpS16*	0.000000	0.003980	0.005180
petL	0.000000	0.001100	0.002980	*rpS18*	0.000000	0.002600	0.002810
petN	0.000000	0.001170	0.003180	*rps19*	0.021620	0.004530	0.004030
psaA	0.093576	0.000890	0.001400	*rpS2*	0.241627	0.001250	0.001210
psaB	0.163367	0.001540	0.001820	*rpS3*	0.307191	0.002370	0.003470
psaC	0.000000	0.002810	0.002330	*rpS4*	0.140003	0.002470	0.002830
psaI	0.000000	0.000000	0.000000	*rpS7*	0.000000	0.000450	0.001220
psaJ	0.037774	0.006260	0.008870	*rpS8*	0.078369	0.001990	0.002830
psbA	0.114451	0.002200	0.002160	*ycf1*	0.584244905	0.004930	0.005980
psbB	0.000000	0.000660	0.000940	*ycf15*	0.000000	0.000000	0.000000
psbC	0.088545	0.001250	0.001560	*ycf2*	0.420059	0.001010	0.001130
psbD	0.000000	0.001410	0.001620	*ycf3*	0.257943	0.002150	0.001690
psbE	0.000000	0.001580	0.002270	*ycf4*	0.216835	0.001260	0.001550
psbF	0.000000	0.000000	0.000000				

Note: *π*, nucleotide polymorphism; *θ*, nucleotide polymorphism per site.
